# Taxonomy, comparative genomics of Mullein (*Verbascum*, Scrophulariaceae), with implications for the evolution of *Verbascum* and Lamiales

**DOI:** 10.1186/s12864-022-08799-9

**Published:** 2022-08-08

**Authors:** Xiang Dong, Elijah Mbandi Mkala, Elizabeth Syowai Mutinda, Jia-Xin Yang, Vincent Okelo Wanga, Millicent Akinyi Oulo, Victor Omondi Onjolo, Guang-Wan Hu, Qing-Feng Wang

**Affiliations:** 1 CAS Key Laboratory of Plant Germplasm Enhancement and Specialty Agriculture, Wuhan Botanical Garden, Chinese Academy of Sciences, Wuhan, 430074 China; 2 Sino-Africa Joint Research Center, Chinese Academy of Sciences, Wuhan, CN-430074 China; 3 University of Chinese Academy of Sciences, Beijing, CN-100049 China; 4 East African Herbarium, National Museums of Kenya, P.O Box 451660-0100, Nairobi, Kenya

**Keywords:** *Verbascum*, Phylogenetic, Plastome, Comparative genomics, Divergence time

## Abstract

**Background:**

The genus *Verbascum* L. (Scrophulariaceae) is distributed in Africa, Europe, and parts of Asia, with the Mediterranean having the most species variety. Several researchers have already worked on the phylogenetic and taxonomic analysis of *Verbascum* by using ITS data and chloroplast genome fragments and have produced different conclusions. The taxonomy and phylogenetic relationships of this genus are unclear.

**Results:**

The complete plastomes (cp) lengths for *V. chaixii*, *V. songaricum*, *V. phoeniceum*, *V. blattaria*, *V. sinaiticum*, *V. thapsus,* and *V. brevipedicellatum* ranged from 153,014 to 153,481 bp. The cp coded 114 unique genes comprising of 80 protein-coding genes, four ribosomal RNA (rRNA), and 30 tRNA genes. We detected variations in the repeat structures, gene expansion on the inverted repeat, and single copy (IR/SC) boundary regions. The substitution rate analysis indicated that some genes were under purifying selection pressure. Phylogenetic analysis supported the sister relationship of (Lentibulariaceae + Acanthaceae + Bignoniaceae + Verbenaceae + Pedaliaceae) and (Lamiaceae + Phyrymaceae + Orobanchaceae + Paulowniaceae + Mazaceae) in Lamiales. Within Scrophulariaceae, *Verbascum* was sister to *Scrophularia,* while *Buddleja* formed a monophyletic clade from (*Scrophularia* + *Verbascum*) with high bootstrap support values. The relationship of the nine species within *Verbascum* was highly supported.

**Conclusion:**

Based on the phylogenetic results, we proposed to reinstate the species status of *V. brevipedicellatum* (Engl.) Hub.-Mor. Additionally, three genera (*Mazus*, *Lancea,* and *Dodartia*) placed in the Phyrymaceae family formed a separate clade within Lamiaceae. The classification of the three genera was supported by previous studies. Thus, the current study also suggests the circumscription of these genera as documented previously to be reinstated. The divergence time of Lamiales was approximated to be 86.28 million years ago (Ma) (95% highest posterior density (HPD), 85.12–89.91 Ma). The complete plastomes sequence data of the *Verbascum* species will be important for understanding the *Verbascu*m phylogenetic relationships and evolution in order Lamiales.

**Supplementary Information:**

The online version contains supplementary material available at 10.1186/s12864-022-08799-9.

## Introduction

Scrophulariaceae family, generally known as the “figwort” consist of angiosperm plants that are herbs and with one genus of shrubs [1]. The family has about 62 genera and approximately 1830 recognized species [2]. The phylogenetic relationship within Scrophulariaceae remains unresolved topic in angiosperm systematics [3]. To date, only chloroplast plastomes of three genera; *Buddleja* L. [4], *Scrophularia* L. [5], and *Verbascum* L. [6, 7], have been studied in this family. The genus *Verbascum* (Scrophulariaceae, Lamiales), commonly recognized as “Mullein”, comprises about 360 species that are widely distributed in temperate regions in Europe, Africa, and Asia [8–11]. The main diversity hotspot for this genus is Turkey with 235 existing species [12]. The genus *Verbascum* is still poorly understood, and new species are described regularly, particularly in Turkey [13]. The taxonomy of *Verbascum* has been a source of contention, and it varies depending on the treatment. This genus is morphologically characterized by rodulate, biennial, or perrenial herbs with yellow-flowered, thyrsic, or racemose inflorescences [1, 14]. *Verbascum* extracts, decoctions, and infusions have been utilized in traditional medicine for a long time. The leaves have been used as a diuretic, sudorific, expectorant, sedative and the flowers have mucolytic and expectorant properties [15–17].

The generic circumscriptions, and placement of the genus *Verbascum* is a challenge [18], and this has been the focus of contentious debate among the researchers. The initial description and classification of *Verbascum* was done by Linnaeus [19], grouped species with 4 stamens in the genus *Celsia* L. and species with 5 stamens in the genus *Verbascum*. However, Fischer and Meyer [20] separated *Verbascum nacolicum* to a new genus *Staurophragm*a because of the oblong cylindrical capsule, a distinguishable feature that he observed. In addition, species which were grouped under the genus *Celsia* were considered as *Scrophularia* by wilder [21], *Allonsona* by (Rui and Pav. 1786), *Janthe* and *Thapsandra* by Griseb., in 1844, *Triguera* by Dunal (1852), and lastly *Alects* by Schintz (1889). Bentham’s treatment adopted the classification of *Celsia* and *Verbascum* by Linneaus’s, based on the presence of stamens [22]. Kuntze (1891) combined the genus *Celsia* with *Verbascum,* based on overall resemblance [23]. However, Murbeck (1925) distinguished *Celsia* and *Verbascum* by the number of stamens, the presence of a sessile or stipitate placenta, and the number of blooms in each bract [24]. Distinguishing the species based on morphological features (four or five stamens) was found unreliable. Thus, two genera *Celsia* and *Staurophragma* were transferred to *Verbascum* [18, 25, 26]. The findings of molecular phylogenetic investigations later verified the classification [13]. *Verbascum's* systematics has had little attention in contemporary to Scrophulariaceae research; hence, it isone of the least understood in terms of infrageneric categorization. Murbeck (1933), separated this genus into two sections using seed morphology, *Aulacospermae* Murb. with a longitudinally grooved seed, and *Bothrospermae* (Murb.) Kamelin with diagonally grooved and alveolate seeds [27, 28]. Huber-Morath (1978) presented an alternate classification system for *Verbascum* in his review of the Turkish members of the genus [29]. In his treatment of the genus, he erected 13 artificial groups A to M among 243 *Verbascum* species (including 129 hybrids) and claimed that all Turkish *Verbascum* species belonged to sect. Bothrospermae Murb. In another regional analysis published for the U.S.S.R separated *Verbascum* and *Celsia* and documented 51 species [28]. In 1981, another regional classification published for the Iranian plateau, merged the genus *Celsia* with *Verbascum*, but the classification did not suggest any infrageneric classification for the forty-nine species and 4 additional hybrids [29], while classification for Europe documented nearly a hundred species of *Verbascum*, including *Celsia* [25].

For the *Verbascum* species distributed in East Africa, one species is only recognized from the region *V. brevipedicellatum* and one new genus *Pelidium* from the family Scrophulariaceae was documented [30–32], while *V. sinaiticum* is distributed in Africa and Arabian Peninsula. *Verbascum brevipedicellatum* is a perennial herb that is well defined morphologically [31]. Currently, the regional flora of Somalia and plants of the world online database (https://powo.science.kew.org/) shows the infrageneric classification of the *V. brevipedicellatum* first published in 1973 was a synonym of *Rhabdotosperma brevipedicellata* (Engl.) Hartl [33–35]. However, in the Flora of Tropical East Africa, *R. brevipedicellat*a is documented as a synonym of *V. brevipedicellatum* with a note description that it is an extremely variable species, and field studies would be necessary to study the morphological variations among populations. Currently, with the material studied, it was difficult to distinguish the species, even to classify them to lower infraspecific ranks that have been described around this species. Though documented as a synonym of *R. brevipedicellata* in the plants of the world online (https://powo.science.kew.org/)*.* It is still unclear if the recognition of this species is warranted or supported by other lines of evidence, such as phylogenetic data*.*

Recently, molecular analyses have been conducted to understand the phylogeny of the genus *Verbascum*. The first study was conducted using a nuclear internal transcribed spacer (ITS) and three non-coding chloroplast DNA regions (*trnY-T*, *psbA-trnH*, and *trns-G*) using 41 taxa representing two different genera [13]. This study supported the monophyly of the genus *Verbascum*, which agreed with the previous morphological studies of Scrophulariaceae [27]. However, the study established no supportive evidence for the subgeneric classification of the genus *Verbascum*. Previuous molecular analysis conducted by Ghahremaninejad et al. [13] suggested that the genus could be monophyletic. Sotoodeh [36], further investigated the genus in Iran and his finding was similar. However, recent studies using plastomes of *V. phoeniceum* L. and *V. chinense* L. and twenty-eight species in the order Lamiales revealed the placement of the genus *Verbascum* [6, 7]. Bi et al. [6], showed that *Verbascum* formed a monophyletic branch sister to *Scrophularia* while another study by He et al. [7], placed *Verbascum* as sister to *Scrophularia*. *Buddleja* formed a sister clade to (*Verbascum* + *Scrophularia*). Besides, the study by He et al. [7], was not in agreement with the previous studies which indicated the monophyly of the genus. Therefore, the classification of this genus within the family Scrophulariaceae is controversial, and more molecular data is required to solve the existing problem. In comparison to chloroplast DNA markers, plastome studies provide more comprehensive genetic data [5, 37, 38]. Plastomes have been recently used as a useful tool in phylogenetic studies and genetic diversity [39–41]. Additionally, the variable regions of plastomes can be used as molecular markers for future phylogenetic and genetic diversity studies [41]. In this study, we sequenced and analyzed the whole plastomes of *V. sinaiticum*, *V. brevipedicellatum,V. thapsus*, *V. songaricum*, *V. blattaria*, *V. chaixii, V. phoeniceum* including two additional sequences from NCBI (NC050920 and MT610040). The main goals of this study are (i) to annotate and compare the plastomes of the eight species of genus *Verbascum*; (ii) to confirm if the genus *Verbascum* is monophyletic or nested within *Scrophularia; (iii)* to evaluate the infraspecific classification of *Verbascum brevipedicellatum* to *Rhabdotosperma brevipedicellatum* (Engl.) Hartl using phylogenetic analysis; (iv) to identify the fast-evolving cpDNA markers for species identification, phylogenetic construction, and phylogeographic studies in future; (v) to understand the evolutionary relations of *Verbascum* species and other species in order Lamiales.

## Results

### Plastomes features

**Fig. 1:**
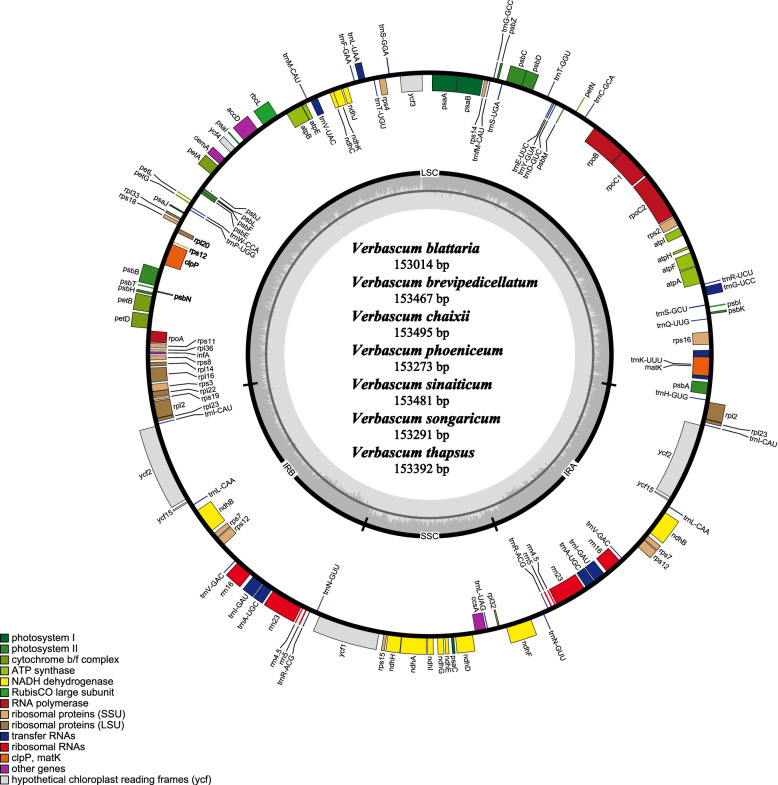
The complete chloroplast plastomes circular map of the *Verbascum sinaiticum, V. thapsus,* and *V. brevipedicellatum*. The gene's direction outside the circle map is transcribed counter-clockwise while those inside the circular map are transcribed clockwise. The colored bars represent diverse functional parts. The dark gray inner circle represents the guanine-cytosine (GC) content, while the light-gray inner circle represents the AT content of the plastome

Genome sequencing was performed using the Illumina paired-end technology platform at the Novogene Company (Beijing, China), the total genomic DNA of seven (one accession (*V. phoenecium*) is similar to the one available in the NCBI) *Verbascum* species were sequenced (Table 1). The plastomes of these seven species were typical circular double-stranded structures and ranged from 153,014 bp in *V. blattaria* to 153,291 bp in *V. chaixii* (Fig. 1). The plastome sequences of the seven *Verbascum* species were deposited in GenBank (Table 1). All seven plastomes show a quadripartite structure, including LSC region (84,263 -84,833 bp) and SSC region (17,811—17,884 bp), which are separated by a pair of IRs (25,426- to 25,467 bp) regions. Comparative analysis among the seven species plastomes showed that *V. chaixii* was the largest in size compared to others. All plastomes encoded 114 unique genes, comprising 80 protein-coding genes, 30 tRNA genes, and four rRNA genes (Fig. 1, Table 2).

**Table 1:** Voucher number, collection place, pairs of reads used and average base-coverage of the sequenced species

species	Voucher number	Collection	Pairs of reads used	Average base-coverage
*V. chaixii*	CPG-73024	Xinjiang, China	3,784,286	543.2
*V. songaricum*	CPG-72889	Xinjiang, China	7,697,759	444.7
*V. sinaiticum*	SAJIT-003840	Mt. Kenya, Kenya	13,682,874	519.1
*V. thapsus*	HGW-2029	Yunan, China	4,096,754	431.5
*V. brevipedicellatum*	SAJIT-001330	Lake Nkuga, Kenya	6,223,865	492.3
*V. phoeniceum*	CPG-72157	Xinjiang, China	4,475,832	430.6
*V. blattaria*	CPG-73143	Xinjiang, China	5,470,460	1,742.6

**Table 2:** Gene composition in seven *Verbascum* species ‡Genes comprising two introns. †Genes with a single intron. Ψ Pseudogene. (× 2), genes duplicated in the inverted repeat regions

Category of Genes	Group of Genes	Name of genes
Self-replication	DNA dependent RNA polymerase	*rpoA, rpoB, rpoC1*^†^*, rpoC*2
	Ribosomal proteins (SSU)	*rps*11*,rps*12*, rps*14*, rps*15*, rps*16^†^*, rps*18*, rps*19*, rps*2*, rps*3*, rps*4*, rps*7, *rps*7*, rps*8
	Ribosomal proteins (LSU)	*rpl*14, *rpl*16^†^*, rpl*2^†^*, rpl*20*, rpl*22*, rpl*23*, rpl*23*, rpl*32, *rpl*33, *rpl*36
RNA genes	Ribosomal RNA	*rrn* 4.5(× 2)*, rrn*5(× 2*), rrn*16(× 2)*, rrn*23(× 2)
	Transfer RNA	*trnH-GUG, trnK-UUU*^†^*, trnQ-UUG, trnS-GCU, trnG-UCC, trnR-UCU, trnC-GCA, trnfM-CAU, trnD-GUC, trnY-GUA, trnE-UUC, trnT-GGU, trnS-UGA, trnG-GCC, trnG-UCC*^†^*, trnS-GGA, trnT-UGU, trnL-UAA*^†^*, trnF-GAA, trnV-UAC*^†^*, trnM-CAU, trnW-CCA, trnP-UGG, trnI-CAU*(× 2), *trnL-CAA*(× 2), *trnV-GAC*(× 2), *trnI-GAU*^†^ (× 2*), trnR-ACG, trnL-UAG, trnN-GUU*(× 2*), trnR-ACG*(× 2*), trnA-UGC* ^†^ (× 2*),*
Genes for photosynthesis	Subunits of ATP synthase	*atpA, atpB, atpE, atpF*^†^*, atpH, atpI*
	Subunits of photosystem I	*psaA, psaB, psaC, psaI, psaJ*
	Subunits of photosystem II	*psbA, psbB, psbC, psbD, psbE, psbF, psbI, psbJ, psbK, psbL, psbM, psbN, psbT, psbZ*
	Subunits of NADH-dehydrogenase	*ndhA*^†^*,, ndhB*^†^ (× 2*), ndhC, ndhD, ndhE, ndhF, ndhG, ndhH, ndhI, ndhJ, ndhK*
	Subunits of cytochrome b/f complex	*petA, petB*^†^*, petD*^†^*, petG, petL, petN*
	Subunit of rubisco	*rbcL*
Other genes	Maturase	*matK*
	Protease	*clpP*^**‡**^
	Subunit of Acetyl-CoA-carboxylase	*accD*
	c-type cytochrome synthesis gene	*ccsA*
	Envelop membrane protein	*cemA*
Genes of unknown function/Hypothetical protein RF	Conserved open reading frames	*ycf*1*, ycf*15 Ψ*, ycf*2*, ycf*4*, ycf*3^**‡**^

The IR regions contained 20 duplicated genes including nine protein-coding genes (*rpl*12*, rpl*23*, ycf*2, *ycf*15*, ndhB, rps*7*, rps*12*, rps*19*, and rpI*22*)*, seven tRNA genes (*trnH-GUG*, *trnI-CAU*, *trnL-CAA*, *trnA-UGC*, *trnR-ACG*, *trnN-GUU, and trnV-AC*) and four rRNAs (*rrn*4.5*, rrn*5*, rrn*16*, rrn*23). The SSC region contained 13 genes of which 12 were CDs and one tRNA, while the LSC region contained 59 protein-coding genes and 22 tRNA genes (Fig. 1). In total, 15 genes (*rps16, atpF, rpoC1, petB, petD, rpl16, rpl2, ndhB, ndhA, trnI-GAU, trnA-UGC, trnV-UAC, trnL-UAA, trnG-UCC, trnK-UUU*) had one intron with *rpl2* and *ndhB* duplicated in the IR, whereas two genes, *clpP* and *ycf3* had two introns. The *rps*12 gene was trans-spliced; the 3’ intron and exon were duplicated in the inverted repeat region and the 5’-exon was found in the large single-copy region (Table S1). The total GC content for seven *Verbascum* species was 38% similar to the previous two published *Verbascum* species (*Verbascum phoeniceum* (NC_050920) and *Verbascum chinense* (MT610040) (Table 3) [6, 7]. In the IR guanine content was 43.2% much higher when compared to LSC 36.1%, and SSC 32.3% region, similar to a study performed under the family Oleaceae [42].

**Table 3:** Comparison of all *Verbascum* species characteristics

Feature	*V. sinaiticum*	*V. thapsus*	*V. brevipedicellatum*	*V. chaixii*	*V. songaricum*	*V. phoeniceum*	*V. blattaria*	*V. phoenecium* (NC_050920)	*V. chinense* (MT610040)
Genome size	153,481	153,392	153,467	153,495	153,291	153,273	153,014	153,348	153,618
Large single copy	84,715	84,666	84,678	84,779	84,534	84,571	84,263	84,601	84,834
Small single copy	17, 832	17,844	17,811	17,844	17,839	17,850	17,871	17,791	17,884
Inverted repeat	25,467	25,441	25,444	25,436	25,459	25,426	25,440	25,478	25,474
**A-T Percentage**									
Guanine content %	38	38	38	38	38	38	38	38	38
LSC	36.1	36	36	36	36	36	36	36	36
SSC	32.3	32.3	32.3	32.3	32.2	32.3	32.3	32.3	32.3
IR	43.2	43.2	43	43	43	43	43	43	43
Number of genes	80	80	80	80	80	80	80	80	80
Number of tRNAs	30	30	30	30	30	30	30	30	30
Number of rRNAs	4	4	4	4	4	4	4	4	4
Duplicated genes	20	20	20	20	20	20	20	20	20

### Repeat analysis

**Fig. 2:**
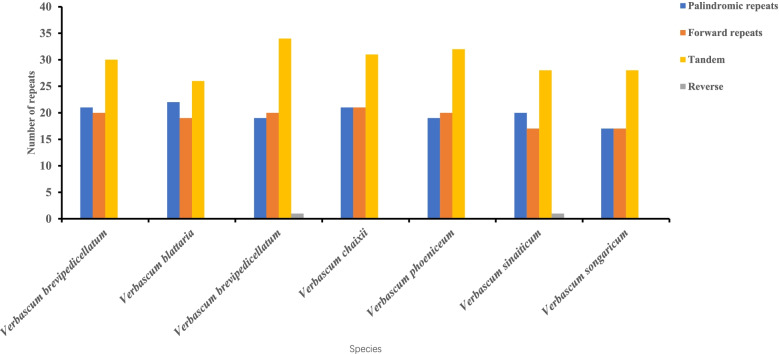
Total number of repeats found in *V. sinaiticum, V. brevipedicellatum,* and *V. thapsus*

Repeat sequences in the seven sequenced *Verbascum* species were analyzed using Tandem and REPuter. A total of 209 tandem repeats were detected in all seven *Verbascum* species (Fig. 2). The number of tandem repeats was 30 in *V. blattaria*, 26 in *V. brevipedicellatum*, 34 in *V. chaixii*, 31 in *V. phoeniceum*, 32 in *V. sinaiticum*, 28 in *V. songaricum*, and 28 in *V. thapsus* (Fig. 2). A total of 264 long repeats had a length of 30–41 base pairs, 7 motifs with 44 base pairs, a length of 52 base pairs, and a length of 61 base pairs was found in the long repeats analysis. *Verbascum blattaria* and *V. brevipedicellatum* each contained 41 long repeats with 21 palindromic (P), and 20 forward (F) (Fig. 2, Table S2-S3). *Verbascum chaixii* had 40 long repeats comprising of 19 palindromic (P),20 forward (F), and 1 reverse (R), and had the longest repeats sequence of 61 bp (Fig. 2, Table S4). *Verbascum phoeniceum* and *V. sinaiticum* each recorded 42 and 39 repeats. Of these, 21 were palindromic (P), 21 forward (F) long repeats, and 19 palindromic (P), 20 were forward (F) long repeats, respectively (Fig. 2. Table S5-6). *Verbascum songaricum* was found to have 38 long repeats, of which 20 were palindromic (P), 17 were forward (F), and 1 was reverse (R) (Fig. 2, Table S7). *Verbascum thapsus* had only a minimum of 34 long repeats, of which both palindromic (P) and forward (F) had 17 each, and it had a longer 52 bp long repeat (Fig. 2, Table S9). No complement repeats were found in any of these species, except for one reverse repeat sequence identified in each of *V. chaixii* and *V. songaricum*. Repeats regions were found in four genes in all seven species: *psaB*, *psaA*, *ycf2*, and *ycf3*. The number of tandem repeats was 30 in *V. blattaria*, 26 in *V. brevipedicellatum*, 34 in *V. chaixii*, 31 in *V. phoeniceum*, 32 in *V. sinaiticum*, 28 in *V. songaricum*, and 28 in *V. thapsus* (Fig. 2).

**Fig. 3:**
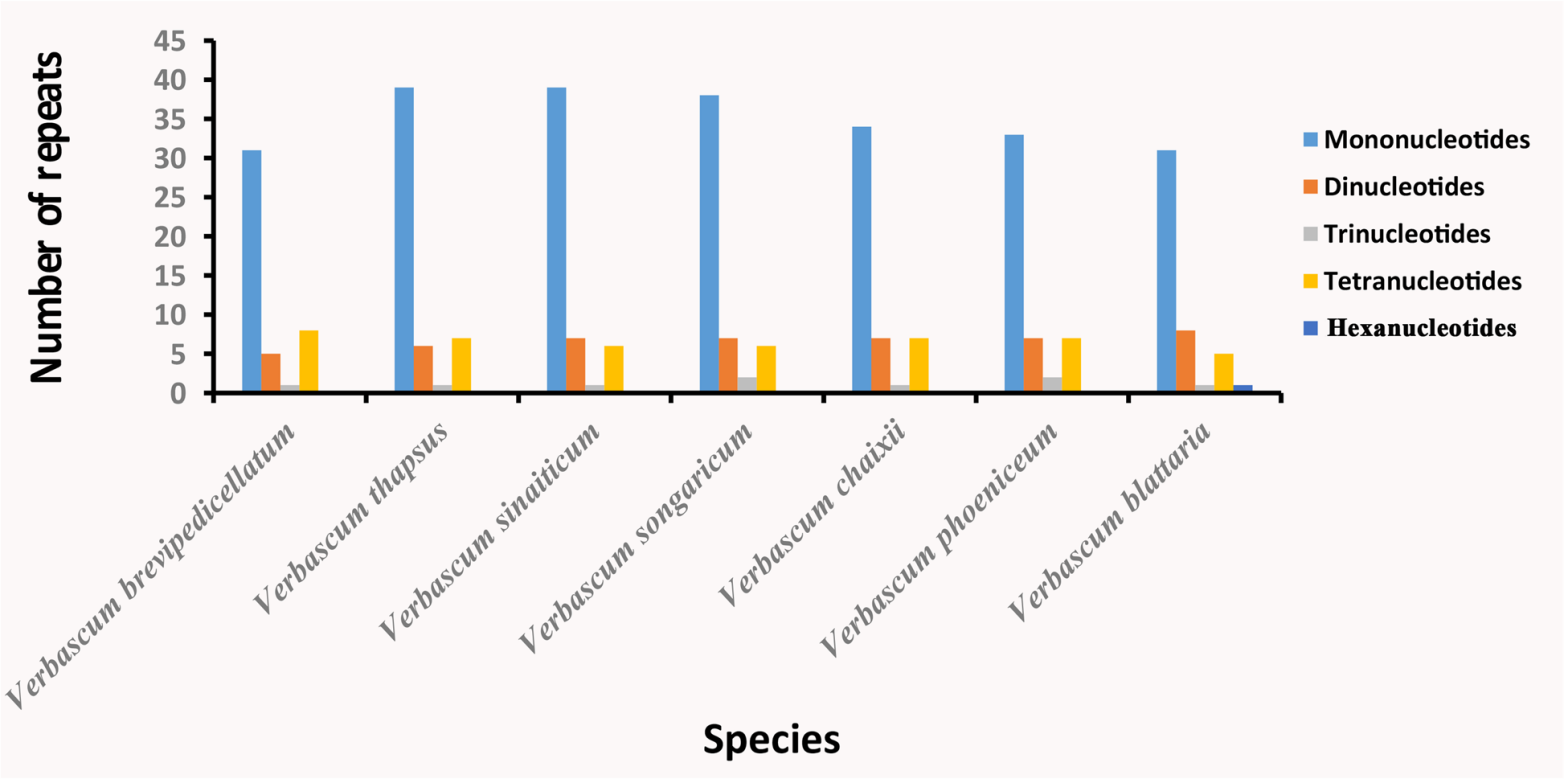
Total number of SSRs repeats present in *V. sinaiticum, V. brevipedicellatum,* and *V. thapsus*

In the MISA analyses, a total of 348 microsatellites regions (> = 10 bp) were identified in the seven plastomes (Table 2). Each *Verbascum* species plastome was found to contain 45–53 SSRs, of which 53 SSRs were shared among the three plastomes, 49 SSRs in two species. No pentanucleotides motifs were found in seven *Verbascum* species while hexanucleotide motifs were only found in *V. blattaria*. Among these repeats, mononucleotides motifs were the most abundant and most SSRs contributed to the AT richness in all seven *Verbascum* species (Fig. 3; Table S9-S10). Most of the motifs were found to be located in the non-coding regions and within ycf2 of the *Verbascum* followed by SSC and IR regions (Table S9). A high resemblance pattern was discovered when comparing the types of SSRs in the seven species. SSRs found in the plastomes of seven *Verbascum* species are generally comparable. These SSRs could be employed as molecular markers for species discrimination, genetic diversity, and evolution research.

### Comparative analysis

The *Verbascum* plastomes divergence results showed that IR regions have higher similarity compared to the single copy (SC) regions. More genes were conserved in the coding regions than in the noncoding regions, which is a common phenomenon in most angiosperms [42]. Significantly, the most conserved regions were observed across all species in the tRNA and rRNA regions. High variation was observed in the intergenic spacer regions of *trnH-GUG—psbA, atpH – atpI, rps16 – trnQ-UUG, petN -psbM, psaA – ycf3, ycf4 – cemA*. Non-coding regions in *ccsA – psaC, rpl32—trnL-UAG* reported high divergence in the SSC. The coding regions with the highest variation include *accD*, *rpoC2* and *matK*. Divergence was also detected in introns of *trnK-UUU, rps16, ycf3, petD, rpl16, clpP, rps12* and *ndhA*. These are regions of rapid evolutionary changes and therefore are essential sites for the development of molecular markers that could be useful in population genetics and phylogenetic studies.

### Divergence hotspot identification

**Fig. 4:**
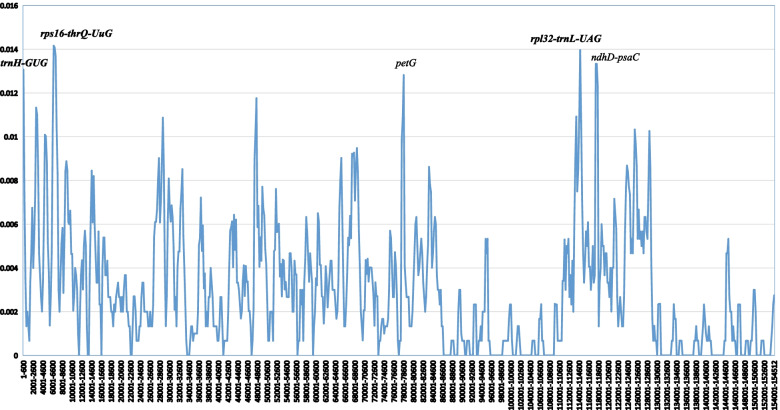
Sliding window analysis based on the 5 cp genome sequences within *Verbascum*. X-axis: position of the Midpoint of a window; Y-axis: Nucleotide diversity of each window (Pi)

To estimate the divergence of among the *Verbascum* species, a total of 5 cp genome sequences of *Verbascum* were chosen to calculate nucleotide diversity (Pi)**,** including *V. sinaiticum, V. thapsu, V. brevipedicellatum, V. phoeniceum* and*V. chinense.* Based on this analysis, we identified five remarkably divergent regions among the fiveplastomes, which were higher than the 0.012 Pi value (*rps16-trnQ-UUG, rpl32-trnL-UAG, ndhD-psaC, trnH-GUG,* and *petD* (Fig. 4)). Gene *trnH-GUG* was the most divergent region with the highest Pi value of (0.013) and is located in the LSC region. These highly divergent regions could be used as potential molecular markers for phylogenetic reconstruction of the genera *Verbascum*. Overall, the result of this study revealed that sequence divergence was concentrated in the LSC and SSC regions, whereas IR regions presented less divergence, consistent with the mVISTA results (Fig. S1).

### Inverted repeats

**Fig. 5:**
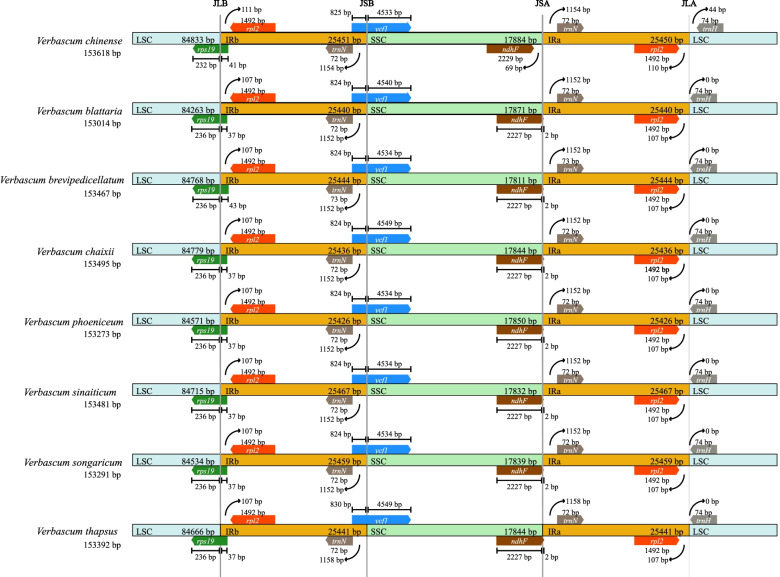
Comparison of the LSC, IR, and SSC boundaries between *Verbascum* cp plastomes

The eight *Verbascum* plastomes compared LSC/IRs and IRs/SSC borders and their adjacent genes (Fig. 5). The *rps*19 gene was located in all *Verbascum* species in the LSC/IRb region. In *V. brevipedicellatum rps*19 gene prolonged to the IR with 43 bp and *V, chinense* with 41 bp. While in the other remaining six *Verbascum* species *rps19* gene extended with 37 bp in length (Fig. 5). The *ycf*1 gene was situated at the IRb/SSC border junction, extending 824 bp into the IRb region in all *Verbascum* species. The *ndhF* gene was situated at the junction of SSC/IRa, extending 2 bp into the IRa region. Overall, the structure and gene content of the eight plastomes s were consistent, no significant expansion or contraction of IR regions were found in the *Verbascum* species.

### Selective pressure analysis

Rates of synonymous (Ks) and non-synonymous (Ks) substitution rates were calculated using a total of 119 regions extracted (coding and non-coding regions) from the plastomes of *V. sinaiticum*, *V. brevipedicellatum,* and *V. thapsus* as follows: 78 protein-coding genes, four rRNAs, and 37 tRNAs with intergenic spacer locations. In all the extracted regions, the Ka and Ks values of *trnD-*trnY (3.75887, 3.24071) and *rpoc*1 gene (3.76133, 3.24071) were the highest. The Ka/Ks was also evaluated because it is used to determine the effect of selective pressure imposed on specific genes. The Ka/Ks value of 15 extracted regions (*atpI, atpH, CssA, matK, ndhE, psaJ, psbE, psbN, psbT*, *rpl*23, *rps*14, *rps*19, *ycf*15, and *trnH-psbA*) was above 1 indicating positive selection (adaptive evolution) while the rest of all the other genes were less than 1 indicating purifying selection in the genes, suggesting that there has been evolutionary pressure to conserve the ancestral state (negative selection), (Fig. S2) (Ka/Ks = 1, neutral selection, Ka/Ks < 1, purifying selection and Ka/Ks > 1 positive selection) [43]. Purifying selection is common in many protein-coding regions [44]. In this study, 90% of the genes had a Ka/Ks ratio of less than 1. Among the extracted regions, the genes with the highest Ka/Ks variability can be used as candidate barcodes to differentiate among *Verbascum* species and in the future applied to perform phylogenetic and phylogeography analysis.

To further verify the results, we calculated the non-synonymous (dN) and synonymous (dS) substitution rates. dN/dS method was used to compare in the selection analysis to check for putative bias for the same functional protein-coding sequences in 80 genes of seven species, including *V. brevipedicellatum*, *V. sinaiticum*, *V. thapsus*, *V. phoeniceum*, *V. chinense*, *B. colvilei*, and *S. henryi* in EasyCodeML software [45]. The ratio (ω = dN/dS) were calculated based on four site-specific models (M0 vs. M3, M1a vs. M2a, M7 vs. M8, and M8a vs. M8) with likelihood ratio test (LRT) threshold of *p* < 0.05 elucidating adaptation signatures within the genome. Among the four models, the comparative LRT of M7 vs. M8 was positive in determining the chi-square *p*-value < 0.05 and the selection strength. Bayes Empirical Bayes (BEB) [46] analysis was implemented in model M8, two sites were detected as the site of positive selection which represented one photosynthesis-related gene *ndhF*, and hypothetical gene ycf1 (Table 4).

**Table 4:** Positively selected sites were detected in the cp genome of the Scrophulariaceae

Gene Name	M8	
	**Selected Sites**	**Pr (w > 1)**
*ycf1*	380 K	0.954*
	846 L	0.953*
*ndhF*	7951 S	0.957*
	7954 L	0.955*

### Phylogenetic analysis

To gain a further insight into the phylogenetic position within *Verbascum* species and their relationship to other closely related species and other families in order Lamiales, two data sets including 80 coding sequence (CDs) genes and nrDNA (ITS1 + 5.8S + ITS2) sequences were aligned to construct the phylogenetic tree.

**Fig. 6:**
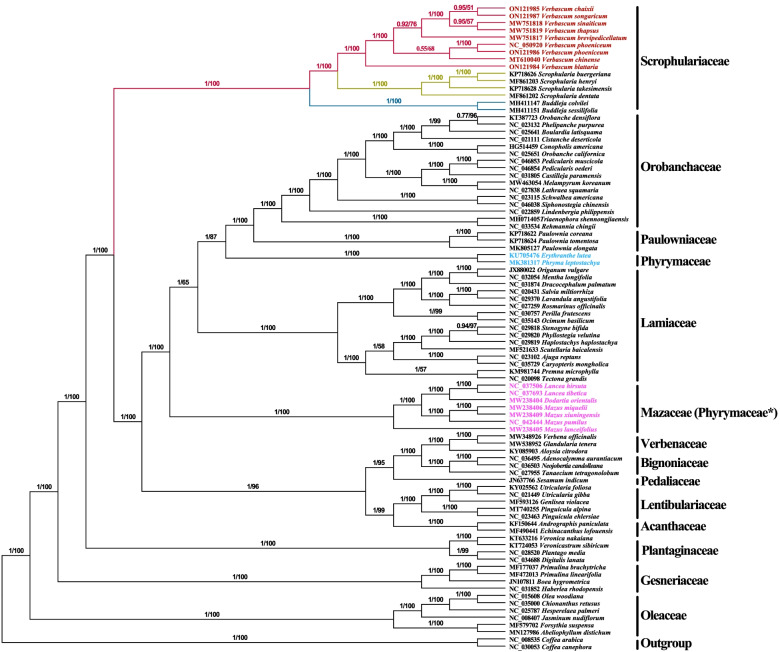
Phylogenetic tree construction of 89 taxa using maximum likelihood (ML) and Bayesian inference (BI) methods using 80 protein-coding genes

**Fig. 7:**
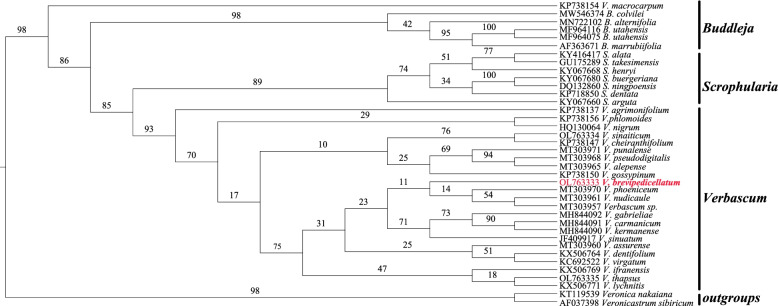
Phylogenetic tree construction of 38 taxa using maximum likelihood (ML) in nrDNA (ITS1 + 5.8S + ITS2)

A phylogenetic study using 80 coding sequence (CDs) genes formed a well-supported tree. The Maximum Likelihood (ML) and Bayesian Inference (BI) inferences produced similar trees with a high support value (95% of nodes with bootstrap support > 90 in ML, 97% of nodes with bootstrap support > 0.9 in BI) (Fig. 6). The families, Scrophulariaceae, Oleaceae, Gesneriaceae, and Plantaginaceae were the first basal angiosperms of Lamiales which branched early. Within the higher core, Lamiales (Lentibulariaceae + Acanthaceae + Bignoniaceae + Verbenaceae + Pedaliaceae) formed a sister clade with (Lamiaceae + Mazaceae + Orobanchaceae + Paulowniaceae + Phyramaceae) families (Mazaceae noted with steric because currently is treated differently within Phyramaceae and in our study, it formed a sister clade to Lamiaceae). Within Scrophulariaceae, *Verbascum* formed a sister clade to *Scrophularia,* while the genus *Buddleja* formed a monophyletic clade. Within *Verbascum*, *V. blattaria* formed a sister clade to *(V. chinense* + *V. phoeniceum*) + (*V. brevipedicellatum* + *V. thapsus* + *V. sinaiticum* + *V. songaricum* + *V. chaixii*) with high bootstrap support values indicating they are closely related and belong to the same genera. In addition, the phylogenetic analysis based on the nrDNA (ITS1 + 5.8S + ITS2) sequence within Scrophulariaceae (*Verbascum* + *Scrophularia* + *Buddleja*) suggested that *V. brevipedicellatum* formed a sister clade to *V. phoeniceum* + (*V. nudicaule* + *Verbascum sp*.) with high bootstrap support values indicating they are closely related and belong to the genus *Verbascum* (Fig. 7).

### Divergence time estimation

**Fig. 8:**
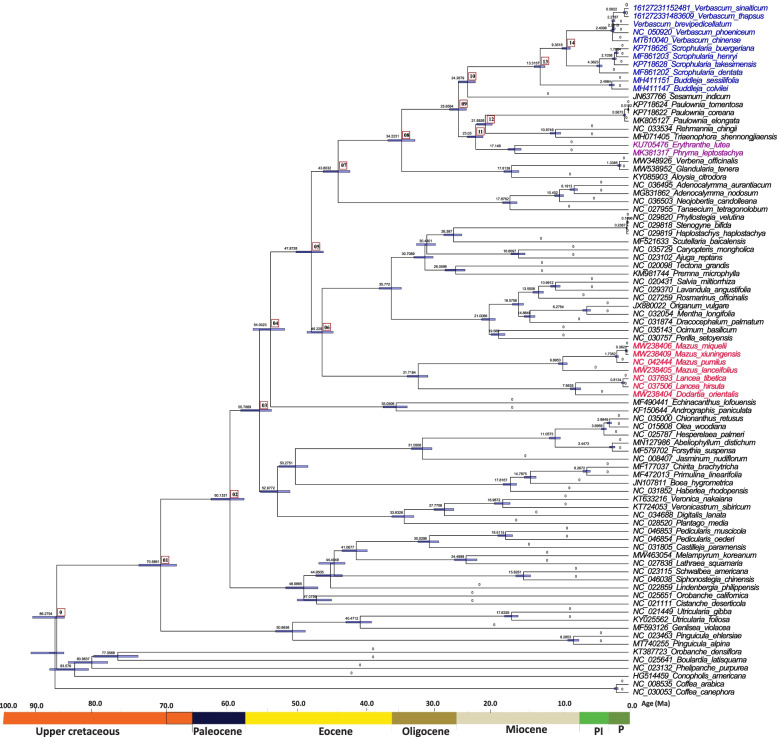
Phylogenetic chronogram showing the evolutionary dating time of order Lamiales using 86 taxa. The tree was estimated using Bayesian analysis of 80 protein-coding genes in the MCMC tree. The number in the circle in red relates to our two nodes of interest

The divergence time of Lamiales (node 0) was approximated at 86. 28 million years ago (Ma) (HPD% 85.12–89.76) (Fig. 8). The Orobanchaceae species diverged at different clades, the first clade diverged at an estimated 83.578 Ma (node 0), same as Lamiales, (95% HPD 85.12- 89.76), which relates closely with the cretaceous junction. The second clade diverged at an estimated 60.13 MA (node 2), (95% HPD 58.01–62.98) which relates closely with Paleocene-Eocene junctions, the third clade diverged at 21.59 Ma (node 12), (95% HPD 20.54–22.88) from Paulowniaceae. Scrophulariaceae diverged at 24.26 Ma (node 10) (95% HPD 23.10- 25.67) from Pedaliaceae. Moreover, within the family Scrophulariaceae, *Buddleja* was estimated to diverge at 13.32 Ma *(95%* HPD% 12.59–14.21) sister to *Scrophularia* and *Verbascum,* while *Verbascum* diverged at 9.35 Ma (95% HPD 8.77- 10.00) sister to *Scrophularia.* Whereas within *Verbascum* the four species diverged at 2.41 Ma (95% HPD 2.17–2.65. However, Phyramaceae and Mazaceae species diverged at different times, Phyramaceae species (*Erythranthe lutea* + *Phryma leptostachya)* diverged at an estimated 23.03 Ma (node 11), (95% HPD 20.54–22.88) sister to Paulowniaceae and Orobanchaceae which relates with the Oligocene and Miocene junctions. While Mazaceae diverged at an estimated 46.23 Ma (node 6), (95% HPD 44.55–48.49) sister to Lamiaceae.

## Discussion

### Comparative plastome analysis

Plastomes are used in taxonomic and evolutionary studies to assess evolutionary relationships and compare genome structure, particularly closely related species**.** Comparative study of *Verbascum* plastomes indicated that they are highly conserved. The complete plastomes of seven *Verbascum* species were quadripartite in structure and composed of the LSC, SSC, and (IRa/IRb) repeat regions [47, 48]. The plastome sizes of seven *Verbascum* species ranged from 153,014 to 153,481 bp. The total gene content and arrangement of seven species of *Verbascum* were almost similar. The seven species confined the equal number of CDs, rRNA, and tRNA genes. Due to the low substitution rate in their genomes and their recent estimated time divergence, this kind of conservatism is common. Previous studies performed in different closely related taxa, for example, tribes and genera have reported similar findings [49, 50].

The complete plastomes of most angiosperm plants are highly conserved, but contraction and expansion of the SC and IR regions are believed to be the major cause of plastome size variations [51, 52]. For instance, inversions, loss of genes, absence of IR region, contraction/expansion in IR, and duplication of the *rps*19 gene in the IR have been reported in different species [53, 54]. In this study, we observed that the boundary region between the two inverted repeats and single copy region were highly conserved, and gene distribution and location specification were consistent. Additionally, a comparison of genes near the IR region of eight different *Verbascum* species showed that genes exhibit different degrees of contraction/expansion at the boundary of the IR region. These results are consistent with previous conclusions, which showed that most angiosperm plants plastomes are usually highly conserved, but little differences occur due to contraction and expansion of the IR and SC junction regions [55–57].

### Repeat analysis

Normally, most repeats are found dispersed in noncoding regions and within the *ycf*2 gene [58]. Comparative analyses of different complete plastomes have indicated that repeated motifs are the factors that cause gene deletion, insertion, and replacement [59, 60]. The repeat analyses of the seven *Verbascum* species detected 264 repeats, most of which are 30–41 bp long, with 7 repeats of 44 bp, 52 bp, and 61 long. Among the seven *Verbascum* species, *V. chaixii* contained the largest number of repeated sequences. Plastome combination and sequence differences mostly occur because of untimely recombination of repetitive sequences and slipped-strand mismatches [56]. These repeats are the basis of genetic markers for phylogenetic and population analyses, being applied widely because of their highly polymorphic and simplicity to be amplified [61, 62]. In this analysis, 348 simple sequence repeat motifs were found, most of them were poly-A and T as previously supported by other studies [63, 64]. In addition, dispersed repeats have been increasingly recognized as a potential genetic variation and regulation source. The repeats found in seven *Verbascum* species indicate genetic variation among the species. Together with these regulatory roles, a structural role of repeated DNA in shaping the 3D folding of genomes has also been proposed [65].

### Selective pressure analysis

The synonymous (Ks) and non-synonymous (Ka) substitution rates and their corresponding ratio (Ka/Ks) also known as (dN/dS) have been used widely in calculating nucleotide evolution rates and natural selection pressure [46]. Generally, studies done previously indicated that Ka/Ks ratios mostly are lower than one [66], due to synonymous nucleotide substitutions rates that occur more often compared to nonsynonymous substitutions rates. The genes with the highest Ka/Ks variability can be used as candidate barcodes to differentiate species and in the future applied to perform phylogenetic and phylogeographic analyses. In this study, 15 extracted regions (*atpI, atpH, CssA, matK, ndhE, psaJ, psbE, psbN, psbT*, *rpl*23, *rps*14, *rps*19, *ycf*15, and *trnH-psbA*) were above 1 indicating positive selection.

To further confirm the results, we calculated the non-synonymous (dN) and synonymous (dS) substitution rates using EasyCodeML [45] of seven species, including *V. brevipedicellatum, V. sinaiticum, V. thapsus, V. phoeniceum, V. chinense, B. colvilei, S. henryi* within Scrophulariaceae species. We identified two sites that were detected as the positive selection site, which represented one photosynthesis-related gene *ndhF*, and hypothetical gene *ycf1* (Table 4). All 17 sites can be used to differentiate species and applied in phylogenetic and phylogeography analyses of the genus *Verbascum.* The varying results of the Ka/Ks ratio obtained in our study are evidence that evolutionary rates of chloroplast genomes vary among genes. This supported by the previous conclusions drawn by Manezes et al., (2018) [67].

### Phylogenetic analysis

The sequencing of complete plastomes has increased recently due to the advancement in sequencing technology [68]. Plastomes are important in molecular, phylogenetic, and evolutionary studies as well as solving relationships in plants and can be applied to plants DNA barcodes [69]. The phylogenetic relationships within Scrophulariaceae is challenging to taxonomists, these difficulties in phylogenetic reconstruction of Scrophulariaceae are likely due to reticulate evolution caused by hybridization and polyploidization [5, 13]. Phylogenetic analysis using 80 protein-coding genes produced a well-supported phylogenetic tree (Fig. 6). The first basal angiosperm classification of Lamiales (Oleaceae, Gesneriaceae, Plantaginaceae, and Scrophulariaceae) was confirmed, and the other families (Phyrymaceae + Orobanchaceae + Paulowniaceae) which were in concordance with the previous studies [5, 70–73]. However, the most problematic of the families’ placement in the previous studies were (Lentibulariaceae + Verbenaceae + Schelegeliaceae + Bignoniaceae + Pedaliaceae + Acanthaceae + Thormandersiaceae + Martyniaceae), which had a support value of (BIPP/MLBS 1/48), but the clade was unsolved in the analyses by Schäferhoff et al. [70], however, a study by Rodriguez & Olmstead et al. [73] found that these families formed a separate clade with support values (BIPP/MLBS/MPBS = 1/91/ < 5, 0.77/25/ < 5, 0.99/58/12). Moreover, Xu et al., [5] study was in agreement with the placement of these families ((Bignoniaceae + Verbenaceae) + Pedaliaceae) + (Acanthaceae + Lentibulariaceae) with a study done by Schäferhoff et al. [70], with high clade support values of (BIPP/MLBS = 1/97) using 80 protein-coding genes. In a study by Bi et al. [6] and He et al. [7], using complete cp genomes, the families formed a clade that was congruent with Xu et al. [5]. However, this study using 80 protein-coding genes with 89 taxa to infer the placement of these families (Lentibulariaceae + Acanthaceae) + Pedaliaceae) formed a sister branch to (Verbenaceae + Bignoniaceae) with high bootstrap clade value of (ML/BI 96/0.971). This may be attributed to fewer data used previously to infer the placement of these families. Moreover, different methods give different topologies, and the genome sequence variation within different species may result in this difference. Within all higher core, Lamiales (Lentibulariaceae + Acanthaceae + Bignoniaceae + Verbenaceae + Pedaliaceae) formed a sister clade to (Lamiaceae + Phyrymaceae + Orobanchaceae + Paulowniaceae + Mazaceae* (*Mazus*, *Lancea,* and *Dodartia*) families, (Mazaceae noted with steric because currently it is treated separately differently within Phyramaceae, in this study, it formed a sister clade with Lamiaceae). The results of this study confirm the results of a previous study that formed a separate clade [74], and consistent with the AGP IV classification of the Mazaceae family [75]. Therefore, in this study, we suggest the classification of the Mazaceae family to be reinstated back to formal recognition as suggested by James L. Reveal [76]. Interestingly, the relationships of the genera within the family Scrophulariaceae is well solved and confirmed.

In the plastomes of 80 CDS tree species, within *Verbascum*, *V. blattaria* formed a sister clade to (*V. chinense* + *V. phoeniceum*) + (*V. brevipedicellatum* + *V. thapsus* + *V. sinaiticum* + *V. songaricum* + *V. chaixii*) with high bootstrap support values indicating they are closely related and belong to the same genera. But the species from Kenya and China were not well separated into two monophyletic lineages. Even two species from Kenya, *V. sinaiticum* and *V. brevipedicellatum*, were clustered with species from China, *V. sinaiticum* was instead more closely related to *V. thapsus* from Yunnan Province, China (Fig. 6). This suggests that research on the origin and biogeography of *Verbascum* species is still underexplored. In addition, the phylogenetic analysis based on nrDNA (ITS1 + 5.8S + ITS2) sequence within Scrophulariaceae (*Verbascum* + *Scrophularia* + *Buddleja*) suggests that *V. brevipedicellatum* formed a sister clade to *V. phoeniceum* + (*V. nudicaule* + *Verbascum sp.*) with high bootstrap support values indicating they are closely related and belong to the genus *Verbascum* (Fig. 7). However, in our study *V. macrocarpum* (KP738154) did not cluster into the genus *Verbascum*, but formed a monophyletic branch, which conforms with the previous study that the species are unresolved [13]. More chloroplast genome data on *V. macrocarpum* may be needed to solve this problem. In this study, our crown age estimation of Lamiales of 86.26 (Ma) (HPD% 85.1–89.9 Ma) was in concordance with the previous studies divergence time estimation (95% HPD, 67-101 Ma, 95% HPD, 70–99.8 Ma) [5, 77–79]. We selected two reliable fossils that represent the old dating period of order Lamiales as constraints for calibrating the age of our angiosperm tree from the well-reported previous analyses [5]. However, Menispermaceae and epiphytic ferns time divergence was estimated around the cretaceous and Paleocene periods which is linked to the development of tropical rain forest angiosperms [80, 81]. The study provides a more current, comprehensive and detailed framework to the evolution of the family Scrophulariaceae and other families in order Lamiales with additional data of complete chloroplast plastomes.

### Taxonomic treatment of Verbascum brevipedicellatum

Our phylogenetic results of nrDNA sequences and plastomes showed *Verbascum brevipedicellatum* formed a sister relationship with other *Verbascum* species. Although several names have been associated with this species in East Africa. In the FTEA it was documented as:

*Verbascum brevipedicellatum* (Engl.) Huber-Morath [family SCROPHULARIACEAE], in Bauhinia 5: 11 (1973); Blundell, Wild Fl. East Afr.: 378, pl. 393 (1987); U.K.W.F.: 254 (1994). Type: Tanzania, Kilimanjaro [Kilimandscharo], Meyer 286 (B†, holo.). Neotype: Tanzania, Kilimanjaro, SE of Bismark hut, Bigger 2012 (K!,)

*Celsia brevipedicellata* Engl. [family SCROPHULARIACEAE], in Abhandl. Akad. Wissensch. Berlin 1891(2): 376 (1892); Skan in F.T.A.: 4(2): 285 (1906); Murb. in Lunds Univ. Arsskr. 22, 1: 65 (1925); A.V.P.: 164 (1957); F.P.U. ed. 2: 133 (1971).

*Celsia brevipedicellata* Murb. var. homostemon [family SCROPHULARIACEAE], in Lunds Univ. Arsskr. 22, 1: 67 (1925). Type: Kenya, Aberdare Mts, 13 March 1922, R.E. & T.C.E. Fries 2262 (UPS!, holo.)

*Celsia brevipedicellata* Murb. var. heterostemon [family SCROPHULARIACEAE], in Lunds Univ. Arsskr. 22, 1: 68 (1925). Type: East Africa, unspecified.

*Celsia keniensis* Murb. [family SCROPHULARIACEAE], in Lunds Univ. Arsskr. 22, 1: 70, t. 2 (1925); Glover, Prov. Check-List Brit. & It. Somal.: 244 (1947). Type: Kenya, Mt Kenya, R.E. & T.C.E. Fries 458 (K!, UPS, syn.), 1869 (UPS!, syn.)

*Celsia floccosa* [family SCROPHULARIACEAE], [sensu Agnew UKWF: 550 (1974), non Benth.]

*Rhabdotosperma brevipedicellata* (Engl.) D. Hartl [family SCROPHULARIACEAE], in Beitr. Biol. Pfl. 53(1): 58 (1977); Fischer, F. A.C. Scrophulariaceae: 12, pl. 2 (1999) & in Fl. Ethiop. & Eritr. 5: 252 (2006).

*Rhabdotosperma keniensis* (Murb.) D. Hartl [family SCROPHULARIACEAE], in Beitr. Biol. Pfl., 53 (1): 58 (1977); Fischer in Thulin (ed.), Fl. Somal.: 3: 266 (2006).

*Verbascum* is a Linnean genus [19], while *Rhabdotosperm*a has been designated by Hartl [82], although in the flora of Somalia *Rhabdotosperma* is the conserved name, so we consider it as superfluous because according to article 14.5 of the International Code of Nomenclature for algae, fungi, and plants [83], specifies that if a conserved name competes with an earlier name against which it has not explicitly been conserved, that the early name is adopted. Therefore, in this regard, *Verbascum* has priority over *Rhabdotosperma* and also over *Celsia*. Additionally, based on the phylogenetic results and type specimens checked, we proposed to reinstate the species status of *Verbascum brevipedicellatum* (Engl.) Hub.-Mor.

## Conclusion

In this study, the plastomes of seven *Verbascum* species (including all the *Verbascum* species distributed in China and two from Kenya) were sequenced and analyzed, determined their phylogenetic placement as well as their dating in the order Lamiales. The chloroplast plastomes indicated that gene organization, GC content, and plastome size are highly conserved. Tandem repeats were highest in *V. chaixii* followed by *V. sinaiticum* and then *V. phoeniceum* (> 31). The other remaining five *Verbascum* species recorded the lower number of tandem repeats (< 31), while simple sequence repeats were highest in *V. chaixii, V. sinaiticum* and then *V. phoeniceum* respectively. The phylogenetic analysis based on 89 taxa indicated that *Verbascum* formed a sister clade to *Scrophularia*. The Scrophulariaceae family diverged at 24.26 Ma (node 9) (95% HPD 23.10—25.67) from Pedaliaceae. The three *Verbascum* species diverged at 9.35 Ma (95% HPD 8.77- 10.00) from *Scrophularia*. Classification of *V. brevipedicellatum* to Rhabdotosperma was not supported by our phylogenetic analysis hence suggesting the reinstatement of the species name. Additionally, we suggest the reinstatement of the families Phyramaceae and Mazaceae because they formed separate clades with high bootstrap support values. Notably, their divergence period was also different; Mazaceae diverged early compared to Phyramaceae species. This study is essential because it indicates the relationship of various families as well as the divergence time estimate in order Lamiales. In addition, phylogenetic results of nrDNA sequences and plastomes supported *Verbascum brevipedicellatum* formed a sister clade with othe *Verbascum* species.

## Materials and methods

### Plant material, DNA extraction, and library preparation

The samples of the seven *Verbascum* species, *V. sinaiticum* (voucher Number SAJIT-003840), *V. brevipedicellatum* (Voucher number SAJIT-001330) were collected from Mt. Kenya and Lake Nkuga, Kenya.*Verbascum chaixii* (voucher Number CPG-73024), *V. songaricum* (voucher Number CPG-72889), *V. phoeniceum* (voucher Number CPG-72157), *V. blattaria* (voucher Number CPG-73143) were collected from Xinjiang province, China, while *V. thapsus* (Voucher number HGW-2029) was collected from Yunnan province, China, and the samples were preserved using Silica gel (Table 1). The voucher specimens were deposited at the East African Herbarium (EA) in the National Museums of Kenya and the Herbarium of Wuhan Botanical Garden, CAS (HIB) (China).

Total genomic DNA was isolated using an improved Cetyltrimenthylammonium bromide (CTAB) method [84]. DNA was quantified through fluorometry using NanoDrop spectrometer or microplate reader, visualized in a 1% agarose-gel electrophoresis for the quality check. A total amount of 1.5 μg DNA per sample was used as input material for the DNA sample preparations. Sequencing libraries were generated using Truseq Nano DNA HT Sample Preparation Kit (Illumina USA) following the manufacturer’s recommendations, and index codes were added to attribute sequences to each sample. Briefly, the DNA sample was fragmented by sonication to a size of 350 bp, then DNA fragments at the end were polished, A-tailed, and ligated with the full-length adapter for Illumina sequencing with further PCR amplification. At last, PCR products were purified (AMPure XP system) and libraries were analyzed for size distribution by Agilent2100 Bioanalyzer and quantified using real-time PCR. Results were then sequenced based on the Illumina paired-end technology platform at the Novogen Company (Beijing, China). In addition to this data, the chloroplast genomes of *Verbascum phoeniceum* (NC_050920) and *Verbascum chinense* (MT610040) and other species used in this study were downloaded from NCBI as well as sequences for phylogenetic analysis (Table 5). The nrDNA (ITS1 + 5.8S + ITS2) used in this study were also downloaded from NCBI (Table 6).

**Table 5:** Species Table Retrieved from NCBI of complete chloroplast genome sequences

Species name	Accession no	References
*Verbascum sinaiticum*	MW751818	The present study assembled
*Verbascum thapsus*	MW751819	The present study assembled
*Verbascum brevipedicellatum*	MW751817	The present study assembled
*Verbascum chaixii*	ON121985	The present study assembled
*Verbascum songaricum*	ON121987	The present study assembled
*Verbascum phoeniceum*	ON121986	The present study assembled
*Verbascum blattaria*	ON121984	The present study assembled
*Verbascum phoeniceum*	NC_050920	Bi et al., 2020 (10.1080/23802359.2020.1715880)
*Verbascum chinense*	MT610040	He et al., 2020 (10.1080/23802359.2020.1715880)
*Genlisea violacea*	MF593126	Silva et al., 2018 (10.1371/journal.pone.0190321)
*Origanum vulgare*	JX880022	Lukas et al., 2013 (10.1016/j.gene.2013.07.026)
*Mentha longifolia*	NC_032054	Vining et al., 2017 (10.1016/j.molp.2016.10.018)
*Dracocephalum palmatum*	NC_031874	Unpublished
*Salvia miltiorrhiza*	NC_020431	Qian et al., 2013 (10.1371/journal.pone.0057607)
*Lavandula angustifolia*	NC_029370	Unpublished
*Rosmarinus officinalis*	NC_027259	Unpublished
*Perilla frutescens*	NC_030757	Unpublished
*Ocimum basilicum*	NC_035143	Unpublished
*Stenogyne bifida*	NC_029818	Unpublished
*Haplostachys haplostachya*	NC_029819	Unpublished
*Phyllostegia velutina*	NC_029820	Unpublished
*Scutellaria baicalensis*	MF521633	Jiang et al., 2017 (10.3390/genes8090227)
*Ajuga reptans*	NC_023102	Zhu et al., 2014 (10.1093/molbev/msu079)
*Caryopteris mongholica*	NC_035729	Liu et al., 2018 (10.1007/s12686-017–0802-5)
*Premna microphylla*	KM981744	Unpublished
*Tectona grandis*	NC_020098	Unpublished
*Mazus miquelii*	MW238406	Unpublished
*Mazus xiuningensis*	MW238409	Unpublished
*Mazus pumilus*	NC_042444	Unpublished
*Mazus lanceifolius*	MW238405	Unpublished
*Dodartia orientalis*	MW238404	Unpublished
*Lancea hirsuta*	NC_037506	Chi et al., 2018 (10.3390/molecules23030602)
*Lancea tibetica*	NC_037693	
*Orobanche densiflora*	KT387723	Cusimano et al., 2016 (10.1111/nph.13784)
*Boulardia latisquama*	NC_025641	Unpublished
*Phelipanche purpurea*	NC_023132	Wicke et al., 2013 (10.1105/tpc.113.113373)
*Orobanche californica*	NC_025651	
*Conopholis americana*	HG514459	
*Lindenbergia philippensis*	NC_022859	
*Pedicularis muscicola*	NC_046853	Unpublished
*Pedicularis oederi*	NC_046854	Unpublished
*Castilleja paramensis*	NC_031805	Unpublished
*Melampyrum koreanum*	MW463054	Unpublished
*Lathraea squamaria*	NC_027838	Samigullin et al., 2016 (10.1371/journal.pone.0150718)
*Schwalbea americana*	NC_023115	Unpublished
*Siphonostegia chinensis*	NC_046038	Gao et al., 2019 (10.1080/23802359.2018.1564384)
*Triaenophora shennongjiaensis*	MH071405	Xia et al., 2018 (10.1080/23802359.2018.1467242)
*Rehmannia chingii*	NC_033534	Zeng et al., 2016 (10.1007/s12686-016–0577-0)
*Paulownia coreana*	KP718622	Yi et al., 2016 (10.1080/23802359.2016.1214546)
*Paulownia tomentosa*	KP718624	
*Scrophularia buergeriana*	KP718626	
*Erythranthe lutea*	KU705476	Vallejo‐Marín et al., 2016 (10.3732/ajb.1500471)
*Phryma leptostachya*	MK381317	Xia et al., 2019 (10.3389/fpls.2019.00528)
*Utricularia foliosa*	KY025562	Silva et al., 2017 (10.1007/s12686-016–0653-5)
*Utricularia gibba*	NC_021449	Ibarra-Laclette et al., 2013 (10.1038/nature12132)
*Verbena officinalis*	MW348926	Unpublished
*Pinguicula alpina*	MT740255	Unpublished
*Pinguicula ehlersiae*	NC_023463	Unpublished
*Andrographis paniculata*	KF150644	Unpublished
*Echinacanthus lofouensis*	MF490441	Unpublished
*Sesamum indicum*	JN637766	Yi et al., 2012 (10.1371/journal.pone.0035872)
*Adenocalymma aurantiacum*	NC_036495	Fonseca et al., 2017 (10.3389/fpls.2017.01875)
*Neojobertia candolleana*	NC_036503	
*Tanaecium tetragonolobum*	NC_027955	Nazareno et al., 2015 (10.1371/journal.pone.0129930)
*Glandularia tenera*	MW538952	Unpublished
*Aloysia citrodora*	KY085903	Unpublished
*Scrophularia henryi*	MF861203	Unpublished
*Scrophularia takesimensis*	KP718628	Unpublished
*Scrophularia dentata*	MF861202	Unpublished
*Buddleja colvilei*	MH411147	Ge et al., 2018 (10.3390/molecules23061248)
*Buddleja sessilifolia*	MH411151	
*Veronica nakaiana*	KT633216	Choi et al., 2016 (10.3359/fpls.2016.00355)
*Veronicastrum sibiricum*	KT724053	
*Plantago media*	NC_028520	Zhu et al., 2016 (10.1111/nph.13743)
*Digitalis lanata*	NC_034688	Unpublished
*Chirita brachytricha*	MF177037	Unpublished
*Primulina linearifolia*	MF472013	Unpublished
*Boea hygrometrica*	JN107811	Zhang et al., 2011 (10.1186/1746–4811-7–38)
*Haberlea rhodopensis*	NC_031852	Unpublished
*Olea woodiana*	NC_015608	Besnard et al., 2011 (10.1186/1471–2229-11–80)
*Chionanthus retusus*	NC_035000	He et al., 2017 (10.1007/s12686-017–0704-6)
*Hesperelaea palmeri*	NC_025787	Unpublished
*Jasminum nudiflorum*	NC_008407	Lee et al., 2007 (10.1093/molbev/msm036)
*Forsythia suspensa*	MF579702	Wang et al., 2017 (10.3390/ijms18112288)
*Abeliophyllum distichum*	MN127986	Min et al., 2019 (10.1080/23802359.2019.1679678)
*Coffea arabica*	NC_008535	Unpublished
*Coffea canephora*	NC_030053	Unpublished

**Table 6:** Species Table Retrieved from NCBI of nrDNA sequences

Species name	Accession no	References
*V. brevipedicellatum*	OL763333	The present study assembled
*V. sinaiticum*	OL763334	The present study assembled
*V. thapsus*	OL763335	The present study assembled
*V. lychnitis*	KX506771	Unpublished
*V. ifranensis*	KX506769	Unpublished
*V. virgatum*	KC692522	Navarro-Perez et al., 2013 (10.1016/j.ympev.2013.05.027)
*V. dentifolium*	JX880022	Unpublished
*V. assurense*	MT303960	Unpublished
*V. sinuatum*	JF409917	Attar et al., 2011 (10.1080/11263504.2011.590826)
*V. kermanense*	MH844090	Sotoodeh et al., 2018 (10.5252/adansonia2018v40a15)
*V. carmanicum*	MH844091	
*V. gabrieliae*	MH844092	
*Verbascum sp.*	MT303957	Unpublished
*V. nudicaule*	MT303961	Unpublished
*V. phoeniceum*	MT303970	Unpublished
*V. alepense*	MT303965	Unpublished
*V. pseudodigitalis*	MT303968	Unpublished
*V. punalense*	MT303971	Unpublished
*V. cheiranthifolium*	KP738147	Ghahremaninejad et al., 2014 (10.1071/Bt14159)
*V. gossypinum*	KP738150	
*V.phlomoides*	KP738156	
*V. agrimonifolium*	KP738137	
*V. macrocarpum*	KP738154	
*S. dentata*	KP718850	Unpublished
*S. ningpoensis*	DQ132860	Unpublished
*S. buergeriana*	KY067680	Scheunert et al., 2017 (10.1007/s13127-016–0316-0)
*S. arguta*	KY067660	
*S. henryi*	KY067668	
*S. takesimensis*	GU175289	Unpublished
*S. alata*	KY416417	Unpublished
*B. marrubiifolia*	AF363671	Schwarzbach et al., 2002 (/)
*B. utahensis*	MF964075	Unpublished
*B. utahensis*	MF964116	Unpublished
*B. alternifolia*	MN722102	Unpublished
*B. colvilei*	MW546374	Culshaw et al., 2021 (10.1002/ajb2.1727)
*Veronica nakaiana*	KT119539	Unpublished
*Veronicastrum sibiricum*	AF037398	Wagstaff et al., 1998 (10.1080/0028825x.1998.9512581)

### Plastome and nrDNA assembly, annotation

The high-quality reads were used for de novo assembly to reconstruct *Verbascum* chloroplast genomes using GetOrganelle v.1.7.2. with wordsize of 150 and K-mer sizes of 105 [85–88]. The visualization of the final assembled graphs was done using Bandage to authenticate the produced plastid plastome [89]. The quality of the newly assembled plastomes was confirmed according to the reading level by aligning the trimmed raw reads to the de novo assemblies using Geneious mapper, Geneious prime 2021 [90], with medium- to low-sensitivity option and iteration up to five times [91]. The annotation of the sequences was performed using CpGAVAS2 [92]. The online blast software version 2.2.25 (https://blast.ncbi.nlm.nih.gov/ Blast. cgi) software was used to match the cp plastomes CDs sequences on NCBI and manual edit them correctly. The tRNAscan-SE software was used to annotate tRNA [93]. The complete circular plastomes were mapped using the OGDRAW program (https://chlorobox.mpimp-golm.mpg. de/OGDraw. html) [94], and the complete genomes were deposited in the Gene bank, Accession numbers: *V. sinaiticum* (MW751818), *V. thapsus* (MW751819), and *V. brevipedicellatum* (MW751817), *V. chaixii* (ON121985), *V. songaricum* (ON121987), *V. phoeniceum* (ON121986) and *V. blattaria* (ON121984). The nrDNA sequence (18S-ITS1-5.8S-ITS2-26S) of *V. sinaiticum* (OL763335), *V. thapsus* (OL763334), and *V. brevipedicellatum* (OL763333) were assembled using GetOrganelle v.1.7.2 with default parameters.

### Repeat analysis

Online REPuter software (https://bibiserv.cebitec.uni-bielefeld.de/reputer) was used to identify and locate forward, palindromic, reverse and complement sequences with minimum repeat size of 20 bp, maximum repeat sequences number of 200 and the E-value below 0.01 [95]. Additionally, tandem repeats were identified using the Tandem repeat finder program [96], with two for the matching alignment and seven for mismatch and indels. Finally, SSRs were identified using the MISA online software (https://webblast.ipk-gatersleben.de/misa/) with the minimum repeat parameters set as 12, 6, 4, 3, 3, 3 repeat units for mono-, di-, tri- tetra-, penta-, and hexanucleotide SSRs, respectively [97].

### Comparative analysis

To compare the structural differences and similarities between *Verbascum* species, the mVISTA online tool was applied to analyze the five representative species (*V. sinaiticum, V. phoeniceum, V. brevipedicellatum, Verbascum chinense, V. thapsus*) plastome sequences with the Shuffle LAGAN model [98], using the reference of the annotation of *V. chinense*. The chloroplast online tool was used to indicate the comparison between the boundaries between, Large Single regions (LSC), Small Single copy (SSC), and the inverted repeats (IR) regions among the eight (*V. sinaiticum, V. phoeniceum, V. brevipedicellatum, Verbascum chinense, V. thapsus, V. chaixii, V. songaricum, V. blattaria*) complete cp plastomes. To explore the highly divergent regions of the cp genomes within eight *Verbascum* species, the software DnaSP version 6.12.03 [99] was used to calculate nucleotide diversity (Pi). The step size and window length were set as 200 bp and 600 bp, respectively. Geneious prime 2021 [90] was used to detect the contraction/expansion of the inverted repeat regions (IRs), and the final graph of expansions/contractions was visualized using Adobe Illustrator.

### Selective pressure analysis

To analyze the substitution rate within *Verbascum* species, a total of 119 regions were extracted from the three representative species (*V. sinaiticum, V. brevipedicellatum, V. thapsus*) cp plastomes. Geneious prime was used to extract the regions. Stop codons were cut and removed manually, and the gaps were removed within the sequences using the Gblocks server online. Mega 7 was used to align combined files after the removal of stop codons and gaps. We converted the aligned files manually by saving them into axt format. The non-synonymous (Ka), synonymous (Ks) substitution rates of 80 protein coding genes and non-coding from the *Verbascum* species and Ka/Ks ratio of each region were calculated using Ka/Ks calculator v 2.0 with maximum likelihood methods Ma (a model that averages parameters across 14 candidate models) and Ms method (a model that has the smallest AICC among 14 candidate models) in a standard code [100].

Another method was employed to dertemine positive selective pressure within shared genes of seven species was evaluated using the PAML v4.7 [45] package implemented in EasyCodeML software. Non-synonymous (dN) and synonymous substitution (dS) substitution rates, and their ratio (ω = dN/dS) were calculated based on four site-specific models (M0 vs. M3, M1a vs. M2a, M7 vs. M8 and M8a vs. M8) with likelihood ratio test (LRT) threshold of *p* < 0.05 elucidating adaptation signatures within the genome. The models permit dN/dS variation within sites while keeping the ω ratio fixed within branches. Selective pressure analysis was conducted along ML tree in plain Newick format based on protein-coding sites used in the generation of phylogenetic relationship of the selected seven species. Here, individual CDS sequences were aligned in correspondence to their amino acids, and their selection was evaluated based on both ω and LRTs values.

### Phylogenetic analysis

To infer the phylogenetic tree, we used 80 protein-coding genes of 87taxa in order Lamiales and two outgroups, which all but seven of our newly sequenced data were downloaded from NCBI. (Table 5), combined in a single file and aligned using MAFFT [101]. Geneious prime version 2021 was used to concatenate the sequence into various readable formats for other analyses. Performed maximum likelihood (ML) analyses using IQ tree [102], integrated with Phylosuite [103], under the best-fitting model according to Akaike Information Criterion (AIC) of all protein-coding genes under the GTR + R6 + F model for 5000 ultrafast bootstraps [104], as well as the Shimodaira–Hasegawa–like approximate likelihood-ratio test [105]. To ascertain the results, BI analysis was performed using the same data in 80 protein-coding genes of 87 taxa in order Lamiales in MrBayes 3.2.7a and using the Markov chain Monte Carlo (MCMC) method, with two independent runs for 10 million generations (Number of Chains is four). We also used second data set to confirm the taxonomic treatment of *Verbascum brevipedicellatum* within the family Scrophulariaceae. We used nrDNA (ITS1 + 5.8S + ITS2) of 36 taxa in the family Scrophulariaceae (*Verbascum* + *Scrophularia* + *Buddleja*), and two outgroups all but three of our newly sequenced data were downloaded from NCBI. (Table 6), sequence alignment was performed using MAFFT v7.308 with default parameters setting. Phylogenetic relationships reconstructions were performed based on maximum likelihood (ML) analysis using the program IQ-Tree with 1000 bootstrap replications. The best-fit model GTR + F + G4 was chosen according to Bayesian Information Criterion (BIC).

### Divergence time estimation

BEAST software was used to perform the analysis. Two secondary calibration fossils and one fossil record were used to constrain the nodes; node (0) the 95% highest posterior density (HPD) the old (limit)divergence time for Lamiales (95% HPD, 67–101 Ma; Barba-Montoya et al. [77]), constraining the crown age of Lamiales (95% HPD, 85.05 Ma (Lognormal priors, 1.0 Mean (M), Sigma (S) 0.5. Constraining the most common ancestor of *Verbascum* (lognormal priors, Off set 65.64, M 1.0, S ¼ 0.5) [5]. The chronogram and branch intervals were linked to partitions and other constraints were unlinked. We used the Yule process prior for speciation and uncorrelated lognormal relaxed clock model. Prior’s constraints time of the nodes were selected using the Log-Normal distribution of mean and standard deviation set at the mean and median limits, and the GTR + I + G substitution model was set as the nucleotide substitution model. Two calibration points were selected to estimate the divergence time of *Verbascum* (Scrophulariaceae): (0) the 95% highest posterior density (HPD) limit of crown age for Lamiales (85.1, 1.0 Mean (M), Sigma (S) 0.5), and to estimate the most common recent ancestor to *Verbascum* and their age divergence (Log-Normal priors, Offset 65.64, M 1.0, S ¼ 0.5). To estimate the dating time, used BEAST2 on XSEDE version 1.8.0 on the CIPRES web server. The MCMC analyses were run for 10 million generations, with sampling every 1000 generations. To check for repeatability, uniformity, and coalescent model parameters in each run three separate BEAST analyses were performed. Tracer version 1.7.1 was used to check the burn-in of trees and chains distribution. Tree Annotator version 1.8.0, was used to summarize and obtain a readable file to show the post-burn-in trees and produce maximum clade credibility. Figtree version 1.4.4 was also used to show the mean divergence time approximates with 95% HPD intervals and to visualize the tree.

## Supplementary Information

**Additional file 1: Fig. S1.** mVISTA percentage identity comparison among five plastomes from Verbascum using V. sinaiticum as reference. X-axis indicates the sequence coordinates in the whole cp genome. Y-axis represents the similarity of the aligned regions, indicating percent identity to the reference genome (50-100%).

**Additional file 2: Fig. S2.** Substitution rate analysis of three Verbascum species, coding DNA sequence (CDS), tRNAs with intergenic spacer, and ribosomal RNA (rRNA).

**Additional file 3: Fig. S3.** Phylogenetic chronogram showing the evolution dating time, 95% highest posterior density (HPD) of the 86 taxa in the order Lamiales.

**Additional file 4: Table S1.** The lengths of introns and exons for the splitting genes. **Table S2.** *Verbascum blattaria* long repeat position table. **Table S3.** *Verbascum brevipedicellatum* long repeat position table. **Table S4.** *Verbascum chaixii* long repeat position table. **Table S5.** *Verbascum phoeniceum* long repeat position table. Table S6. *Verbascum sinaiticum* long repeat position table. **Table S7.** *Verbascum songaricum* long repeat position table. **Table S8.** *Verbascum thapsus* long repeat position table. **Table S9.** SRRs present in *Verbascum* species. **Table S10.** Total number of SSRs repeats.

## Data Availability

All data generated or analyzed during this study are included in this published article and the complete chloroplast genome sequences (nrDNA sequences) of *V. sinaiticum, V. brevipedicellatum, V. thapsus*, *V. chaixii*, *V. songaricum*, *V. phoeniceum*, and *V. blattaria* are deposited in the Genbank with ID no: MW751818 (OL763334), MW751819 (OL763333), MW751817 (OL763335), ON121985, ON121987, ON121986, and ON121984,.respectively. The accession numbers corresponding to the additional datasets used and analyzed in this study can be found in Table 5 and Table 6. These were retrieved from National Center for Biotechnology Information database.

## Abbreviations

IRb/IRb: Inverted repeats –IR
LSC: Large single copy
SSC: Short small single copy
rps: Ribosomal proteins genes
rRNA: Ribosomal RNA genes
tRNA: Transfer RNAs genes
IGS: Intergenic region space
SSRs: Simple sequence repeats
SAJIT: Sino-Africa Joint Investigation Team
SAJOREC: Sino-Africa Joint Research Center
HIB: Herbarium of Wuhan Botanical Garden
PCR: Polymerase chain reaction
dNTPs: Deoxynucleoside triphosphate snynthesis
BSA: Bovine serum albumin
BI: Bayesian Inference
ML: Maximum Likelihood

## References

[1] Fischer E, Kadereit JW (2004). Scrophulariaceae.. Flowering Plants Dicotyledons. The Families and Genera of Vascular Plants.

[2] Christenhusz MJ, Byng JW (2016). The number of known plants species in the world and its annual increase. Phytotaxa.

[3] Judd WS, Olmstead RG (2004). A survey of tricolpate (eudicot) phylogenetic relationships. Am J Bot.

[4] Ge J, Cai L, Bi G-Q, Chen G, Sun W. Characterization of the Complete Chloroplast Genomes of Buddleja colvilei and B. sessilifolia: Implications for the Taxonomy of Buddleja L. Molecules. 2018;23(6):1248. 10.3390/molecules23061248 PMC6100213 29882896

[5] Xu WQ, Losh J, Chen C, Li P, Wang RH, Zhao YP, Qiu YX, Fu CX (2019). Comparative genomics of figworts (Scrophularia, Scrophulariaceae), with implications for the evolution of Scrophularia and Lamiales. J Syst Evol.

[6] Bi Y, Deng P, Liu L (2020). The complete chloroplast genome sequence of purple mullein (Verbascum phoeniceum L.). Mitochondrial DNA Part B.

[7] He Y, Ma Y, Li Z, Liu P, Yuan W (2020). The complete chloroplast genome of Verbascum chinense (L.) Santapau. Mitochondrial DNA Part B.

[8] Hassler M. Synonymic Checklists of the Vascular Plants of the World. In: Bánki O, Roskov Y, Vandepitte L, DeWalt RE, Remsen D, Schalk P, Orrell T, Keping M, Miller J, Aalbu R, Adlard R, Adriaenssens E, Aedo C, Aescht E, Akkari N, Alonso-Zarazaga MA, Alvarez B, Alvarez F, Anderson G, et al, editors. Catalogue of Life Checklist (Version 2021-08-06). 2021.

[9] Georgiev MI, Ali K, Alipieva K, Verpoorte R, Choi YH (2011). Metabolic differentiations and classification of Verbascum species by NMR-based metabolomics. Phytochemistry.

[10] Press BRIT (2018). Mabberley’s Plant-Book: A Portable Dictionary of Plants, their Classification and Uses. J Bot Res Inst Tex..

[11] Riahi M, Ghahremaninejad F. The tribe Scrophularieae (Scrophulariaceae): A Review of Phylogenetic Studies. Hacquetia. 2019;18(2).

[12] Yılmaz G, Dane F (2012). The genus Verbascum L. ın European Turkey. Botanica Serbica.

[13] Ghahremaninejad F, Riahi M, Babaei M, Attar F, Behçet L, Sonboli A (2015). Monophyly of Verbascum (Scrophularieae: Scrophulariaceae): evidence from nuclear and plastid phylogenetic analyses. Aust J Bot.

[14] Oxelman B, Kornhall P, Olmstead RG, Bremer B (2005). Further disintegration of Scrophulariaceae. Taxon.

[15] Lust J B, Tierra M. The Natural Remedy Bible. Simon and Schuster; 2003.

[16] Vogl S, Picker P, Mihaly-Bison J, Fakhrudin N, Atanasov AG, Heiss EH, Wawrosch C, Reznicek G, Dirsch VM, Saukel J (2013). Ethnopharmacological in vitro studies on Austria's folk medicine—an unexplored lore in vitro anti-inflammatory activities of 71 Austrian traditional herbal drugs. J Ethnopharmacol.

[17] Kaur V, Upadhyaya K (2016). Antibacterial activity of Verbascum chinense (Scrophulariaceae) extracts. Int J Curr Microbiol App Sci.

[18] Karavelioğulları F, Aytaç Z (2008). Revision of the genus Verbascum L (Group A) in Turkey. Botany Res J.

[19] Linnaeus C. Species plantarum, vol. II. Stockholm; 1753.

[20] Fischer F, Meyer C (1843). Index Hort Petrop.

[21] Wydler H. Essai monographique sur le genre Scrofularia. Barbezat et Delarue; 1828.

[22] Bentham G, Hooker J (1846). Scrophulariaceae. Prodromus systematis naturalis regni vegetabilis.

[23] Kuntze C. Orchidaceae. Revisio generum plantarum. 1891;2:645–82.

[24] Murbeck S. Monographie der gattung Celsia. Gleerup; 1925.

[25] Ferguson I (1972). Verbascum L. Flora europaea.

[26] JWT. Flora of Turkey and the East Aegean Islands, vol. 3. 1972.

[27] Murbeck SS. Monographie der gattung Verbascum. 1933.

[28] Fedchenko BA. Verbascum L. In: Shishkin BKS, Bobrow EG, editors. Flora U. S. S. R. Izdatel’stvo Akademii Nauk S.S.S.R., Leningrad. 1955;22:132–97.

[29] Huber Morath A. Verbascum. In: Rechinger Kh, Flora Iranica. No: 146-149: 1-50.-Akademische Druck-u. Verlag sanastal. Graz. Austria. Verbascum (Scrophulariaceae). Am J Bot. 1981;84(12):1638–45.

[30] Darbyshire I, Luke Q, Vollesen K (2009). Peplidium (scrophulariaceae): a new generic record for the flora of tropical East Africa. J East Afr Nat Hist.

[31] Bytebier B (2008). Flora of Tropical East Africa. J East Afr Nat Hist.

[32] The Plant List. Version 1.1. Published on the Internet. Verbascum brevipedicellatum. http://www.theplantlist.org/tpl1.1/record/tro-50234498. Accessed 1 Jan 2013.

[33] World Checklist of Selected Plant Families. Facilitated by the Royal Botanic Gardens, Kew. Published on the Internet; http://wcsp.science.kew.org/ Retrieved.

[34] IPNI. International Plant Names Index. Published on the Internet http://www.ipni.org, The Royal Botanic Gardens, Kew, Harvard University Herbaria & Libraries and Australian National Botanic Gardens. Retrieved 03 August 2022.

[35] Thulin M: Flora of Somalia: Volume 3: Royal Botanic Gardens; 2006.

[36] Sotoodeh A (2015). Histoire biogéographique et évolutive des genres Verbascum et Artemisia en Iran à l'aide de la phylogénie moléculaire.

[37] Besnard G (2009). Rubio de Casas R, Christin P-A, Vargas P: Phylogenetics of Olea (Oleaceae) based on plastid and nuclear ribosomal DNA sequences: tertiary climatic shifts and lineage differentiation times. Ann Bot.

[38] Wanga VO, Dong X, Oulo MA, Mkala EM, Yang J-X, Onjalalaina GE, Gichua MK, Kirika PM, Gituru RW, Hu G-W (2021). Complete Chloroplast Genomes of Acanthochlamys bracteata (China) and Xerophyta (Africa)(Velloziaceae): Comparative Genomics and Phylogenomic Placement. Front Plant Sci.

[39] Choi KS, Chung MG, Park S (2016). The complete chloroplast genome sequences of three Veroniceae species (Plantaginaceae): comparative analysis and highly divergent regions. Front Plant Sci.

[40] Ruhsam M, Rai HS, Mathews S, Ross TG, Graham SW, Raubeson LA, Mei W, Thomas PI, Gardner MF, Ennos RA (2015). Does complete plastid genome sequencing improve species discrimination and phylogenetic resolution in Araucaria?. Mol Ecol Resour.

[41] Kyalo CM, Li Z-Z, Mkala EM, Malombe I, Hu G-W, Wang Q-F (2020). The first glimpse of Streptocarpus ionanthus (Gesneriaceae) phylogenomics: Analysis of five subspecies’ chloroplast genomes. Plants.

[42] Wang W, Yu H, Wang J, Lei W, Gao J, Qiu X, Wang J (2017). The complete chloroplast genome sequences of the medicinal plant Forsythia suspensa (Oleaceae). Int J Mol Sci.

[43] Nei M (2000). and S.

[44] Nielsen R. Molecular signatures of natural selection. Annu Rev Genet. 2005;39(1):197–218. 10.1146/annurev.genet.39.073003.112420 16285858

[45] Yang Z (2007). PAML 4: Phylogenetic Analysis by Maximum Likelihood. Mol Biol Evol.

[46] Yang Z, Wong WSW, Rasmus N (2005). Bayes empirical bayes inference of amino acid sites under positive selection. Mol Biol Evol.

[47] Wicke S, Schneeweiss GM, Depamphilis CW, Müller KF, Quandt D (2011). The evolution of the plastid chromosome in land plants: gene content, gene order, gene function. Plant Mol Biol.

[48] Zhang Y, Du L, Liu A, Chen J, Wu L, Hu W, Zhang W, Kim K, Lee S-C, Yang T-J (2016). The complete chloroplast genome sequences of five Epimedium species: lights into phylogenetic and taxonomic analyses. Front Plant Sci.

[49] Yao X, Tang P, Li Z, Li D, Liu Y, Huang H (2015). The first complete chloroplast genome sequences in Actinidiaceae: genome structure and comparative analysis. PLoS ONE.

[50] Munyao JN, Dong X, Yang J-X, Mbandi EM, Wanga VO, Oulo MA, Saina JK, Musili PM, Hu G-W (2020). Complete chloroplast genomes of Chlorophytum comosum and Chlorophytum gallabatense: genome structures, comparative and phylogenetic analysis. Plants.

[51] Luo J, Hou B-W, Niu Z-T, Liu W, Xue Q-Y, Ding X-Y (2014). Comparative chloroplast genomes of photosynthetic orchids: insights into the evolution of the Orchidaceae and development of molecular markers for phylogenetic applications. PLoS ONE.

[52] Rono PC, Dong X, Yang J-X, Mutie FM, Oulo MA, Malombe I, Kirika PM, Hu G-W, Wang Q-F (2020). Initial complete chloroplast genomes of Alchemilla (Rosaceae): comparative analysis and phylogenetic relationships. Front Genet.

[53] Sun Y-x (2013). Moore MJ, Meng A-p, Soltis PS, Soltis DE, Li J-q, Wang H-c: Complete plastid genome sequencing of Trochodendraceae reveals a significant expansion of the inverted repeat and suggests a Paleogene divergence between the two extant species. PLoS ONE.

[54] Lei W, Ni D, Wang Y, Shao J, Wang X, Yang D, Wang J, Chen H, Liu C (2016). Intraspecific and heteroplasmic variations, gene losses, and inversions in the chloroplast genome of Astragalus membranaceus. Sci Rep.

[55] Saina JK, Li Z-Z, Gichira AW, Liao Y-Y (2018). The complete chloroplast genome sequence of the tree of heaven (Ailanthus altissima (mill.)(sapindales: Simaroubaceae), an important pantropical tree. Int J Mol Sci.

[56] Asaf S, Khan AL, Khan A, Al-Harrasi A (2020). Unraveling the chloroplast genomes of two Prosopis species to identify its genomic information, comparative analyses, and phylogenetic relationship. Int J Mol Sci.

[57] Gui L, Jiang S, Xie D, Yu L, Huang Y, Zhang Z, Liu Y (2020). Analysis of complete chloroplast genomes of Curcuma and the contribution to phylogeny and adaptive evolution. Gene.

[58] Nazareno AG, Carlsen M, Lohmann LG (2015). Complete chloroplast genome of Tanaecium tetragonolobum: the first Bignoniaceae plastome. PLoS ONE.

[59] Yi X, Gao L, Wang B, Su Y-J, Wang T (2013). The complete chloroplast genome sequence of Cephalotaxus oliveri (Cephalotaxaceae): evolutionary comparison of Cephalotaxus chloroplast DNAs and insights into the loss of inverted repeat copies in gymnosperms. Genome Biol Evol.

[60] Yao X, Tan Y-H, Liu Y-Y, Song Y, Yang J-B, Corlett RT (2016). Chloroplast genome structure in Ilex (Aquifoliaceae). Sci Rep.

[61] Pauwels M, Vekemans X, Godé C, Frérot H, Castric V, Saumitou-Laprade P (2012). Nuclear and chloroplast DNA phylogeography reveals vicariance among European populations of the model species for the study of metal tolerance, Arabidopsis halleri (Brassicaceae). New Phytol.

[62] Zhao K, Li L, Quan H, Yang J, Zhang Z, Liao Z, Lan X (2021). Comparative analyses of chloroplast genomes from 14 zanthoxylum species: identification of variable DNA markers and phylogenetic relationships within the genus. Front Plant Sci.

[63] Qian J, Song J, Gao H, Zhu Y, Xu J, Pang X, Yao H, Sun C, Li X, Li C (2013). : the complete chloroplast genome sequence of the medicinal plant Salvia miltiorrhiza. PLoS ONE.

[64] Chen J, Hao Z, Xu H, Yang L, Liu G, Sheng Y, Zheng C, Zheng W, Cheng T, Shi J (2015). The complete chloroplast genome sequence of the relict woody plant Metasequoia glyptostroboides Hu et Cheng. Front Plant Sci.

[65] Shapiro JA, Von SR (2010). Why repetitive DNA is essential to genome function. Biol Rev Camb Philos Soc.

[66] Yang J, Kang G-H, Pak J-H, Kim S-C (2020). Characterization and comparison of two complete plastomes of Rosaceae species (Potentilla dickinsii var. glabrata and Spiraea insularis) endemic to Ulleung Island, Korea. Int J Mol Sci.

[67] Apa Menezes (2018). Chloroplast genomes of Byrsonima species (Malpighiaceae): comparative analysis and screening of high divergence sequences. Sci Rep.

[68] Zhou T, Ruhsam M, Wang J, Zhu H, Li W, Zhang X, Xu Y, Xu F, Wang X (2019). The complete chloroplast genome of Euphrasia regelii, pseudogenization of ndh genes and the phylogenetic relationships within Orobanchaceae. Front Genet.

[69] Oulo MA, Yang J-X, Dong X, Wanga VO, Mkala EM, Munyao JN, Onjolo VO, Rono PC, Hu G-W, Wang Q-F (2020). Complete chloroplast genome of Rhipsalis baccifera, the only cactus with natural distribution in the old world: Genome rearrangement, intron gain and loss, and implications for phylogenetic studies. Plants.

[70] SchäFerhoff B (2010). Towards resolving Lamiales relationships: insights from rapidly evolving chloroplast sequences. BMC Evol Biol.

[71] Wortley AH, Rudall PJ, Harris DJ (2005). How much data are needed to resolve a difficult phylogeny? case study in lamiales. Syst Biol.

[72] Albach DC, Yan K, Jensen SR (2009). Phylogenetic placement of Triaenophora (formerly Scrophulariaceae) with some implications for the phylogeny of Lamiales. Taxon.

[73] Refulio-Rodriguez NF, Olmstead RG (2014). Phylogeny of Lamiidae.. Am J Bot..

[74] Chi X, Wang J, Gao Q, Zhang F, Chen S (2018). The complete chloroplast genomes of two Lancea species with comparative analysis. Molecules.

[75] Chase MW, Christenhusz M, Fay M, Byng J, Judd WS, Soltis D, Mabberley D, Sennikov A, Soltis PS, Stevens PF (2016). An update of the Angiosperm Phylogeny Group classification for the orders and families of flowering plants: APG IV. Bot J Linn Soc.

[76] Reveal JL (2011). Summary of recent systems of angiosperm classification. Kew Bull.

[77] Barba-Montoya J, Dos Reis M, Schneider H, Donoghue PC, Yang Z (2018). Constraining uncertainty in the timescale of angiosperm evolution and the veracity of a Cretaceous Terrestrial Revolution. New Phytol.

[78] Li H-T, Yi T-S, Gao L-M, Ma P-F, Zhang T, Yang J-B, Gitzendanner MA, Fritsch PW, Cai J, Luo Y (2019). Origin of angiosperms and the puzzle of the Jurassic gap. Nature plants.

[79] Magallón S, Sánchez-Reyes LL, Gómez-Acevedo SL (2019). Thirty clues to the exceptional diversification of flowering plants. Ann Bot.

[80] Schuettpelz E, Pryer KM (2009). Evidence for a Cenozoic radiation of ferns in an angiosperm-dominated canopy. Proc Natl Acad Sci.

[81] Wang W, Ortiz RDC, Jacques FM, Xiang XG, Li HL, Lin L, Li RQ, Liu Y, Soltis PS, Soltis DE (2012). Menispermaceae and the diversification of tropical rainforests near the Cretaceous-Paleogene boundary. New Phytol.

[82] Hartl D. Rhabdotosperma, eine neue, aus Gliedern von Verbascum L. und Celsia L. gebildete Gattung der Scrophulariaceen. 1977;53, NO 1:55–60.

[83] McNeill J, Barrie F, Buck W, Demoulin V, Greuter W, Hawksworth D, Herendeen P, Knapp S, Marhold K, Prado J. International Code of Nomenclature for algae, fungi and plants (Melbourne Code), vol. 154. Koeltz Scientific Books Königstein; 2012.

[84] Doyle JJ, Doyle JL (1990). Isolation of plant DNA from fresh tissue. Focus.

[85] Jin J-J, Yu W-B, Yang J-B, Song Y, Depamphilis CW, Yi T-S, Li D-Z (2020). GetOrganelle: a fast and versatile toolkit for accurate de novo assembly of organelle genomes. Genome Biol.

[86] Camacho C, Coulouris G, Avagyan V, Ma N, Papadopoulos J, Bealer K, Madden TL (2009). BLAST+: architecture and applications. BMC Bioinformatics.

[87] Bankevich A, Nurk S, Antipov D, Gurevich AA, Dvorkin M, Kulikov AS, Lesin VM, Nikolenko SI, Pham S, Prjibelski AD (2012). SPAdes: a new genome assembly algorithm and its applications to single-cell sequencing. J Comput Biol.

[88] Langmead B, Salzberg SL (2012). Fast gapped-read alignment with Bowtie 2. Nat Methods.

[89] Wick RR, Schultz MB, Zobel J, Holt KE (2015). Bandage: interactive visualization of de novo genome assemblies. Bioinformatics.

[90] Kearse M, Moir R, Wilson A, Stones-Havas S, Cheung M, Sturrock S, Buxton S, Cooper A, Markowitz S, Duran C (2012). Geneious Basic: an integrated and extendable desktop software platform for the organization and analysis of sequence data. Bioinformatics.

[91] Hourahine B, Aradi B, Blum V, Bonafé F, Buccheri A, Camacho C, Cevallos C, Deshaye M, Dumitrică T, Dominguez A (2020). DFTB+, a software package for efficient approximate density functional theory based atomistic simulations. J Chem Phys.

[92] Shi L, Chen H, Jiang M, Wang L, Wu X, Huang L, Liu C (2019). CPGAVAS2, an integrated plastome sequence annotator and analyzer. Nucleic Acids Res.

[93] Schattner P, Brooks AN, Lowe TM (2005). The tRNAscan-SE, snoscan, and snoGPS web servers for the detection of tRNAs and snoRNAs. Nucleic acids research.

[94] Lohse M, Drechsel O, Kahlau S, Bock R (2013). OrganellarGenomeDRAW—a suite of tools for generating physical maps of plastid and mitochondrial genomes and visualizing expression data sets. Nucleic Acids Res.

[95] Kurtz S, Choudhuri JV, Ohlebusch E, Schleiermacher C, Stoye J, Giegerich R (2001). REPuter: the manifold applications of repeat analysis on a genomic scale. Nucleic Acids Res.

[96] Benson G (1999). Tandem repeats finder: a program to analyze DNA sequences. Nucleic Acids Res.

[97] Thiel T, Michalek W, Varshney R, Graner A (2003). Exploiting EST databases for the development and characterization of gene-derived SSR-markers in barley (Hordeum vulgare L). Theor Appl Genet.

[98] Frazer KA, Pachter L, Poliakov A, Rubin EM, Dubchak I (2004). VISTA: computational tools for comparative genomics. Nucleic Acids Res.

[99] Rozas J, Ferrer-Mata A, Sánchez-DelBarrio J C, et al. DnaSP 6: DNA sequence polymorphism analysis of large data sets. Mol Biol Evol. 2017;34(12):3299–302. 10.1093/molbev/msx248 29029172

[100] Li J, Zhang Z, Vang S, Yu J (2009). Wong GK-S, Wang J: Correlation between Ka/Ks and Ks is related to substitution model and evolutionary lineage. J Mol Evol.

[101] Katoh K, Standley DM (2013). MAFFT multiple sequence alignment software version 7: improvements in performance and usability. Mol Biol Evol.

[102] Nguyen L-T, Schmidt HA, Von Haeseler A, Minh BQ (2015). IQ-TREE: a fast and effective stochastic algorithm for estimating maximum-likelihood phylogenies. Mol Biol Evol.

[103] Zhang D, Gao F, Jakovlić I, Zou H, Zhang J, Li WX, Wang GT (2020). PhyloSuite: an integrated and scalable desktop platform for streamlined molecular sequence data management and evolutionary phylogenetics studies. Mol Ecol Resour.

[104] Minh BQ, Nguyen MAT, von Haeseler A (2013). Ultrafast approximation for phylogenetic bootstrap. Mol Biol Evol.

[105] Guindon S, Dufayard J-F, Lefort V, Anisimova M, Hordijk W, Gascuel O (2010). New algorithms and methods to estimate maximum-likelihood phylogenies: assessing the performance of PhyML 30. Syst Biol.

