# Supplementary figures and images for: Taxonomy, comparative genomics of Mullein (Verbascum, Scrophulariaceae), with implications for the evolution of Verbascum and Lamiales
Source: BMC Genomics. 2022 Aug 8;23:566. doi: 10.1186/s12864-022-08799-9 (PMC9358837; doi:10.1186/s12864-022-08799-9)

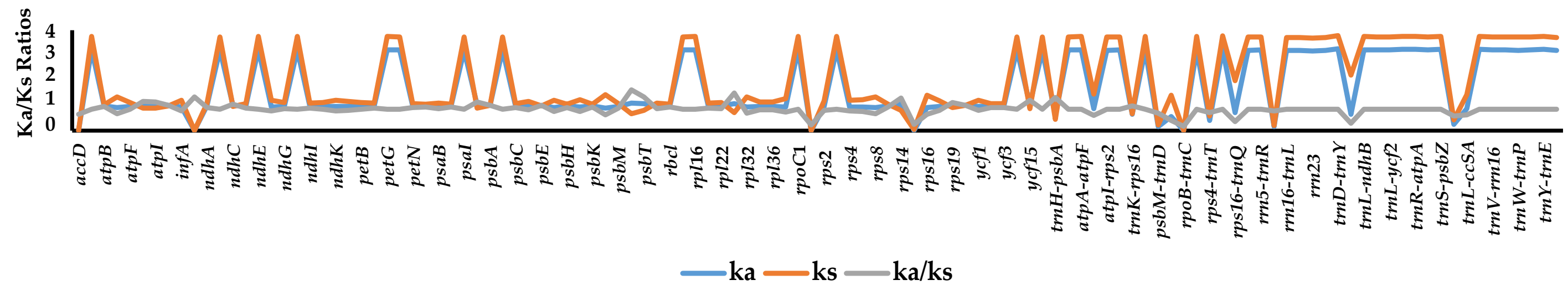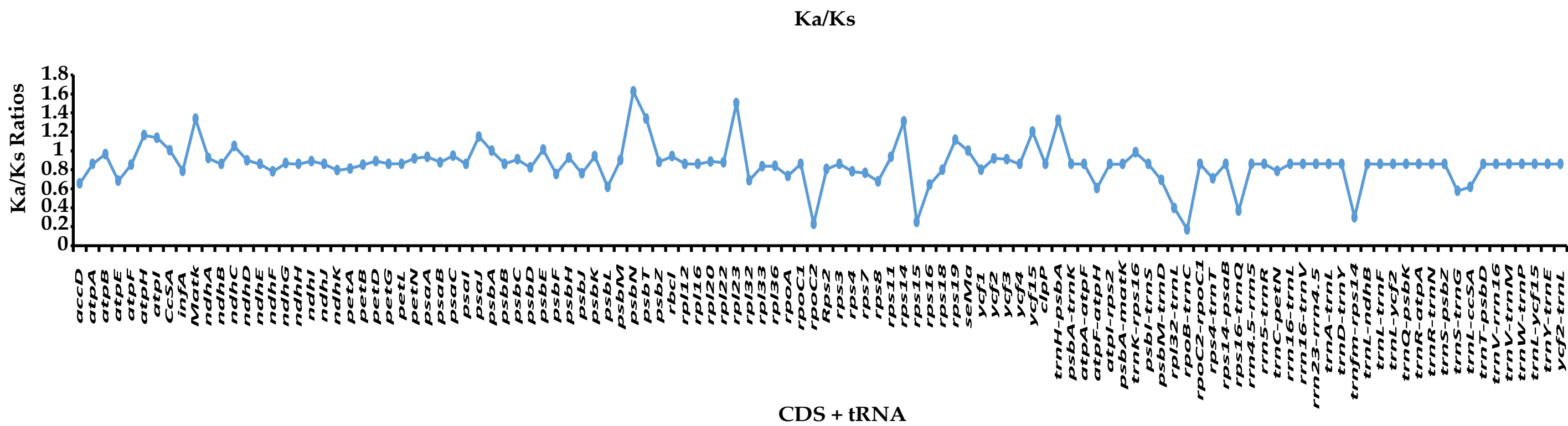

Supplement: Supplementary file 2 — Additional file 2: Fig. S2. Substitution rate analysis of three Verbascum species, coding DNA sequence (CDS), tRNAs with intergenic spacer, and ribosomal RNA (rRNA). [file 12864_2022_8799_MOESM2_ESM.pdf]

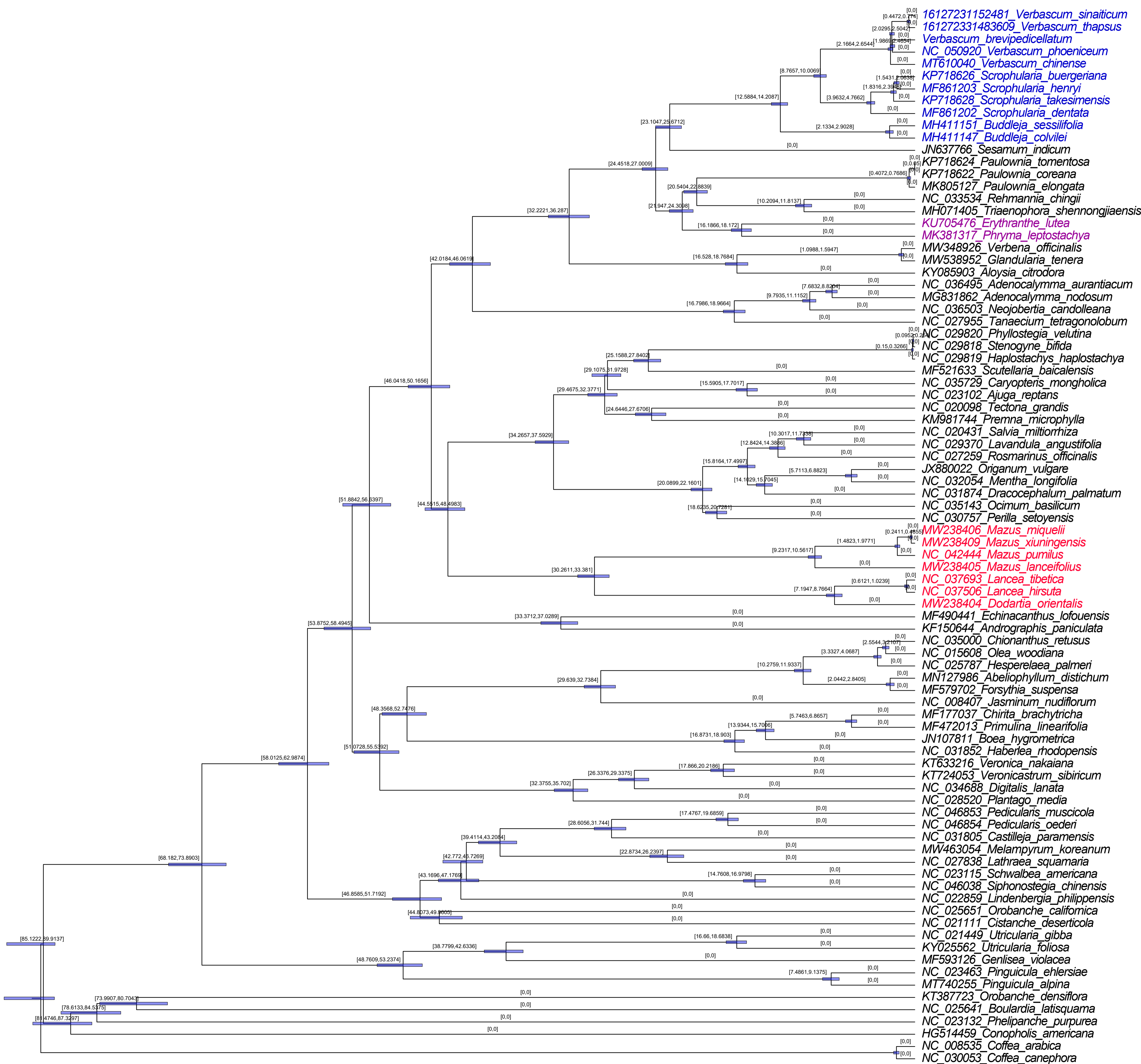

Supplement: Supplementary file 3 — Additional file 3: Fig. S3. Phylogenetic chronogram showing the evolution dating time, 95% highest posterior density (HPD) of the 86 taxa in the order Lamiales. [file 12864_2022_8799_MOESM3_ESM.pdf]
