# Supplementary material for: Taxonomy, comparative genomics of Mullein (Verbascum, Scrophulariaceae), with implications for the evolution of Verbascum and Lamiales
Source: BMC Genomics. 2022 Aug 8;23:566. doi: 10.1186/s12864-022-08799-9 (PMC9358837; doi:10.1186/s12864-022-08799-9)
Supplement: Supplementary file 4 — Additional file 4: Table S1. The lengths of introns and exons for the splitting genes. Table S2. Verbascum blattaria long repeat position table. Table S3. Verbascum brevipedicellatum long repeat position table. Table S4. Verbascum chaixii long repeat position table. Table S5. Verbascum phoeniceum long repeat position table. Table S6. Verbascum sinaiticum long repeat position table. Table S7. Verbascum songaricum long repeat position table. Table S8. Verbascum thapsus long repeat position table. Table S9. SRRs present in Verbascum species. Table S10. Total number of SSRs repeats. [file 12864_2022_8799_MOESM4_ESM.docx]

**Table S1.** The lengths of introns and exons for the splitting genes.

| **Gene** | **Strand** | **Start** | | **End** | | **ExonI** | **IntronI** | **ExonII** | **IntronII** | **ExonIII** |
| --- | --- | --- | --- | --- | --- | --- | --- | --- | --- | --- |
| *trnK-UUU* | - | 1807 | 4389 | | 37 | | 2511 | 35 |  |  |
| *rps16* | - | 4877 | 5994 | | 42 | | 851 | 225 |  |  |
| *trnG-UCC* | + | 9149 | 9905 | | 23 | | 687 | 47 |  |  |
| *atpF* | - | 11871 | 13132 | | 145 | | 707 | 410 |  |  |
| *rpoC1* | - | 20983 | 23793 | | 430 | | 756 | 1625 |  |  |
| *ycf3* | - | 43064 | 45006 | | 129 | | 706 | 228 | 727 | 153 |
| *trnL-UAA* | + | 47976 | 48553 | | 37 | | 491 | 50 |  |  |
| *trnV-UAC* | - | 52435 | 53093 | | 38 | | 584 | 37 |  |  |
| *ClPp* | - | 70787 | 72734 | | 71 | | 734 | 291 | 626 | 226 |
| *petB* | + | 75663 | 77034 | | 6 | | 724 | 642 |  |  |
| *petD* | + | 77212 | 78439 | | 8 | | 745 | 475 |  |  |
| *rpl16* | - | 81864 | 83149 | | 9 | | 878 | 399 |  |  |
| *rpl2* | - | 84823 | 86314 | | 397 | | 664 | 431 |  |  |
| *ndhB* | - | 95122 | 97333 | | 775 | | 679 | 758 |  |  |
| *trnI-GAU* | + | 102660 | 103682 | | 37 | | 951 | 35 |  |  |
| *trnA-UGC* | + | 103747 | 104632 | | 38 | | 813 | 35 |  |  |
| *ndhA* | - | 119375 | 121536 | | 553 | | 1070 | 539 |  |  |
| *trnA-UGC* | - | 133565 | 134450 | | 38 | | 813 | 35 |  |  |
| *trnI-GAU* | - | 134515 | 135537 | | 37 | | 951 | 35 |  |  |
| *ndhB* | + | 140864 | 143075 | | 775 | | 679 | 758 |  |  |
| *rps2* | + | 70784 | 140201 | | 114 | | 114 | 232 | 537 | 26 |

**Table S2.** *Verbascum* *blattaria* long repeat position table

| Repeat size (bp) | 1st start | location1 | | 2st start | location2 | | Repeat type |
| --- | --- | --- | --- | --- | --- | --- | --- |
| 41 | 98511 | *IGS* | (IRb) | 117763 | *ndhA* | (SSC) | P |
| 41 | 117763 | *ndhA* | (SSC) | 138725 | *IGS* | (IRa) | F |
| 44 | 74585 | *IGS* | (LSC) | 74585 | *IGS* | (LSC) | P |
| 39 | 44107 | *ycf3* | (LSC) | 98513 | *IGS* | (IRb) | F |
| 39 | 44107 | *ycf3* | (LSC) | 117763 | *ndhA* | (SSC) | P |
| 39 | 44107 | *ycf3* | (LSC) | 138725 | *IGS* | (IRa) | P |
| 40 | 91497 | *ycf2* | (IRb) | 91533 | *ycf2* | (IRb) | F |
| 40 | 91497 | *ycf2* | (IRb) | 145704 | *ycf2* | (IRa) | P |
| 40 | 91533 | *ycf2* | (IRb) | 145740 | *ycf2* | (IRa) | P |
| 40 | 145704 | *ycf2* | (IRa) | 145740 | *ycf2* | (IRa) | F |
| 30 | 8300 | *trnS-GCU* | (LSC) | 45847 | *trnS-GGA* | (LSC) | P |
| 30 | 42814 | *IGS* | (LSC) | 42843 | *IGS* | (LSC) | F |
| 36 | 91495 | *ycf2* | (IRb) | 91513 | *ycf2* | (IRb) | F |
| 36 | 91495 | *ycf2* | (IRb) | 145728 | *ycf2* | (IRa) | P |
| 36 | 91513 | *ycf2* | (IRb) | 145746 | *ycf2* | (IRa) | P |
| 36 | 145728 | *ycf2* | (IRa) | 145746 | *ycf2* | (IRa) | F |
| 34 | 91515 | *ycf2* | (IRb) | 91533 | *ycf2* | (IRb) | F |
| 34 | 91515 | *ycf2* | (IRb) | 145710 | *ycf2* | (IRa) | P |
| 34 | 91533 | *ycf2* | (IRb) | 145728 | *ycf2* | (IRa) | P |
| 34 | 145710 | *ycf2* | (IRa) | 145728 | *ycf2* | (IRa) | F |
| 31 | 27984 | *IGS* | (LSC) | 28010 | *IGS* | (LSC) | F |
| 30 | 44119 | *ycf3* | (LSC) | 98525 | *IGS* | (IRb) | F |
| 30 | 44119 | *ycf3* | (LSC) | 138722 | *IGS* | (IRa) | P |
| 32 | 8298 | *trnS-GCU* | (LSC) | 35932 | *trnS-UGA* | (LSC) | F |
| 30 | 9881 | *trnG-UCC* | (LSC) | 36812 | *trnG-GCC* | (LSC) | F |
| 30 | 29960 | *IGS* | (LSC) | 29996 | *IGS* | (LSC) | P |
| 30 | 35934 | *trnS-UGA* | (LSC) | 45847 | *trnS-GGA* | (LSC) | P |
| 30 | 39037 | *psaB* | (LSC) | 41261 | *psaA* | (LSC) | F |
| 30 | 44119 | *ycf3* | (LSC) | 117760 | *ndhA* | (SSC) | P |
| 30 | 89083 | *ycf2* | (IRb) | 89125 | *ycf2* | (IRb) | F |
| 30 | 89083 | *ycf2* | (IRb) | 148122 | *ycf2* | (IRa) | P |
| 30 | 89125 | *ycf2* | (IRb) | 148164 | *ycf2* | (IRa) | P |
| 30 | 91496 | *ycf2* | (IRb) | 91550 | *ycf2* | (IRb) | F |
| 30 | 91496 | *ycf2* | (IRb) | 145697 | *ycf2* | (IRa) | P |
| 30 | 91525 | *ycf2* | (IRb) | 91543 | *ycf2* | (IRb) | F |
| 30 | 91525 | *ycf2* | (IRb) | 145704 | *ycf2* | (IRa) | P |
| 30 | 91543 | *ycf2* | (IRb) | 145722 | *ycf2* | (IRa) | P |
| 30 | 91550 | *ycf2* | (IRb) | 145751 | *ycf2* | (IRa) | P |
| 30 | 145697 | *ycf2* | (IRa) | 145751 | *ycf2* | (IRa) | F |
| 30 | 145717 | *ycf2* | (IRa) | 145753 | *ycf2* | (IRa) | F |
| 30 | 148122 | *ycf2* | (IRa) | 148164 | *ycf2* | (IRa) | F |

**Table S3.** *Verbascum brevipedicellatum* long repeat position table

| Repeat size (bp) | 1st start | location1 | | 2st start | location2 | | Repeat type |
| --- | --- | --- | --- | --- | --- | --- | --- |
| 41 | 99012 | *IGS* | (IRa) | 118234 | *ndhA* | (SSC) | P |
| 41 | 118234 | *ndhA* | (SSC) | 139182 | *IGS* | (IRb) | F |
| 44 | 75096 | *IGS* | (LSC) | 75096 | *IGS* | (LSC) | P |
| 40 | 92002 | *ycf2* | (IRb) | 92038 | *ycf2* | (IRb) | F |
| 40 | 92002 | *ycf2* | (IRb) | 146157 | *ycf2* | (IRa) | P |
| 40 | 92038 | *ycf2* | (IRb) | 146193 | *ycf2* | (IRa) | P |
| 40 | 146157 | *ycf2* | (IRa) | 146193 | *ycf2* | (IRa) | F |
| 30 | 8320 | *trnS-GCU* | (LSC) | 45798 | *trnS-GGA* | (LSC) | P |
| 36 | 44075 | *ycf3* | (LSC) | 99017 | *IGS* | (IRb) | F |
| 36 | 44075 | *ycf3* | (LSC) | 118234 | *ndhA* | (SSC) | P |
| 36 | 44075 | *ycf3* | (LSC) | 139182 | *IGS* | (IRa) | P |
| 36 | 92000 | *ycf2* | (IRb) | 92018 | *ycf2* | (IRb) | F |
| 36 | 92000 | *ycf2* | (IRb) | 146181 | *ycf2* | (IRa) | P |
| 36 | 92018 | *ycf2* | (IRb) | 146199 | *ycf2* | (IRa) | P |
| 36 | 146181 | *ycf2* | (IRa) | 146199 | *ycf2* | (IRa) | F |
| 32 | 125468 | *IGS* | (SSC) | 125468 | *IGS* | (SSC) | P |
| 34 | 92020 | *ycf2* | (IRb) | 92038 | *ycf2* | (IRb) | F |
| 34 | 92020 | *ycf2* | (IRb) | 146163 | *ycf2* | (IRa) | P |
| 34 | 92038 | *ycf2* | (IRb) | 146181 | *ycf2* | (IRa) | P |
| 34 | 146163 | *ycf2* | (IRa) | 146181 | *ycf2* | (IRa) | F |
| 30 | 6833 | *IGS* | (LSC) | 6856 | *IGS* | (LSC) | F |
| 30 | 29935 | *IGS* | (LSC) | 29971 | *IGS* | (LSC) | P |
| 30 | 44084 | *ycf3* | (LSC) | 99026 | *IGS* | (IRb) | F |
| 30 | 44084 | *ycf3* | (LSC) | 139179 | *IGS* | (IRa) | P |
| 32 | 8318 | *trnS-GCU* | (LSC) | 35921 | *trnS-UGA* | (LSC) | F |
| 30 | 9902 | *trnG-UCC* | (LSC) | 36798 | *trnG-GCC* | (LSC) | F |
| 30 | 35923 | *trnS-UGA* | (LSC) | 45798 | *trnS-GGA* | (LSC) | P |
| 30 | 39024 | *psaB* | (LSC) | 41248 | *psaA* | (LSC) | F |
| 30 | 44084 | *ycf3* | (LSC) | 118231 | *ndhA* | (SSC) | P |
| 30 | 89588 | *ycf2* | (IRb) | 89630 | *ycf2* | (IRb) | F |
| 30 | 89588 | *ycf2* | (IRb) | 148575 | *ycf2* | (IRa) | P |
| 30 | 89630 | *ycf2* | (IRb) | 148617 | *ycf2* | (IRa) | P |
| 30 | 92001 | *ycf2* | (IRb) | 92055 | *ycf2* | (IRb) | F |
| 30 | 92001 | *ycf2* | (IRb) | 146150 | *ycf2* | (IRa) | P |
| 30 | 92030 | *ycf2* | (IRb) | 92048 | *ycf2* | (IRb) | F |
| 30 | 92030 | *ycf2* | (IRb) | 146157 | *ycf2* | (IRa) | P |
| 30 | 92048 | *ycf2* | (IRb) | 146175 | *ycf2* | (IRa) | P |
| 30 | 92055 | *ycf2* | (IRb) | 146204 | *ycf2* | (IRa) | P |
| 30 | 146150 | *ycf2* | (IRa) | 146204 | *ycf2* | (IRa) | F |
| 30 | 146170 | *ycf2* | (IRa) | 146206 | *ycf2* | (IRa) | F |
| 30 | 148575 | *ycf2* | (IRa) | 148617 | *ycf2* | (IRa) | F |

**Table S4.** *Verbascum* *chaixii* long repeat position table

| Repeat size (bp) | 1st start | location1 | | 2st start | location2 | | Repeat type |
| --- | --- | --- | --- | --- | --- | --- | --- |
| 61 | 42619 | *IGS* | (LSC) | 42679 | *IGS* | (LSC) | F |
| 41 | 99023 | *IGS* | (IRb) | 118251 | *ndhA* | (SSC) | P |
| 41 | 118251 | *ndhA* | (SSC) | 139210 | *IGS* | (IRa) | F |
| 44 | 75107 | *IGS* | (LSC) | 75107 | *IGS* | (LSC) | P |
| 39 | 44187 | *ycf3* | (LSC) | 99025 | *IGS* | (IRb) | F |
| 39 | 44187 | *ycf3* | (LSC) | 118251 | *ndhA* | (SSC) | P |
| 39 | 44187 | *ycf3* | (LSC) | 139210 | *IGS* | (IRa) | P |
| 40 | 92013 | *ycf2* | (IRb) | 92049 | *ycf2* | (IRb) | F |
| 40 | 92013 | *ycf2* | (IRb) | 146185 | *ycf2* | (IRa) | P |
| 40 | 92049 | *ycf2* | (IRb) | 146221 | *ycf2* | (IRa) | P |
| 40 | 146185 | *ycf2* | (IRa) | 146221 | *ycf2* | (IRa) | F |
| 30 | 8310 | *trnS-GCU* | (LSC) | 45911 | *trnS-GGA* | (LSC) | P |
| 35 | 51686 | *IGS* | (LSC) | 51686 | *IGS* | (LSC) | R |
| 36 | 92011 | *ycf2* | (IRb) | 92029 | *ycf2* | (IRb) | F |
| 36 | 92011 | *ycf2* | (IRb) | 146209 | *ycf2* | (IRa) | P |
| 36 | 92029 | *ycf2* | (IRb) | 146227 | *ycf2* | (IRa) | P |
| 36 | 146209 | *ycf2* | (IRa) | 146227 | *ycf2* | (IRa) | F |
| 34 | 92031 | *ycf2* | (IRb) | 92049 | *ycf2* | (IRb) | F |
| 34 | 92031 | *ycf2* | (IRb) | 146191 | *ycf2* | (IRa) | P |
| 34 | 92049 | *ycf2* | (IRb) | 146209 | *ycf2* | (IRa) | P |
| 34 | 146191 | *ycf2* | (IRa) | 146209 | *ycf2* | (IRa) | F |
| 30 | 44199 | *ycf3* | (LSC) | 99037 | *IGS* | (IRb) | F |
| 30 | 44199 | *ycf3* | (LSC) | 139207 | *IGS* | (IRa) | P |
| 32 | 8308 | *trnS-GCU* | (LSC) | 35954 | *trnS-UGA* | (LSC) | F |
| 32 | 16439 | *rps2* | (LSC) | 16440 | *rps2* | (LSC) | F |
| 31 | 16444 | *rps2* | (LSC) | 16444 | *rps2* | (LSC) | P |
| 30 | 9881 | *trnG-UCC* | (LSC) | 36835 | *trnG-GCC* | (LSC) | F |
| 30 | 29951 | *IGS* | (LSC) | 29987 | *IGS* | (LSC) | P |
| 30 | 35956 | *trnS-UGA* | (LSC) | 45911 | *trnS-GGA* | (LSC) | P |
| 30 | 38510 | *psaB* | (LSC) | 40725 | *psaA* | (LSC) | F |
| 30 | 39059 | *psaB* | (LSC) | 41283 | *psaA* | (LSC) | F |
| 30 | 44199 | *ycf3* | (LSC) | 118248 | *ndhA* | (IRb) | P |
| 30 | 64647 | *IGS* | (LSC) | 64671 | *IGS* | (LSC) | F |
| 30 | 89599 | *ycf2* | (IRb) | 89641 | *ycf2* | (IRb) | F |
| 30 | 89599 | *ycf2* | (IRb) | 148603 | *ycf2* | (IRa) | P |
| 30 | 89641 | *ycf2* | (IRb) | 148645 | *ycf2* | (IRa) | P |
| 30 | 92041 | *ycf2* | (IRb) | 92059 | *ycf2* | (IRb) | F |
| 30 | 92041 | *ycf2* | (IRb) | 146185 | *ycf2* | (IRa) | P |
| 30 | 92059 | *ycf2* | (IRb) | 146203 | *ycf2* | (IRa) | P |
| 30 | 146198 | *ycf2* | (IRa) | 146234 | *ycf2* | (IRa) | F |
| 30 | 148603 | *ycf2* | (IRa) | 148645 | *ycf2* | (IRa) | F |

**Table S5.** *Verbascum* *phoeniceum* long repeat position table

| Repeat size (bp) | 1st start | location1 | | 2st start | location2 | | Repeat type |
| --- | --- | --- | --- | --- | --- | --- | --- |
| 41 | 98815 | *IGS* | (IRb) | 119753 | *IGS* | (SSC) | F |
| 41 | 119753 | *IGS* | (SSC) | 138988 | *IGS* | (IRa) | P |
| 44 | 74894 | *IGS* | (LSC) | 74894 | *IGS* | (LSC) | P |
| 39 | 44154 | *ycf3* | (LSC) | 98817 | *IGS* | (IRb) | F |
| 39 | 44154 | *ycf3* | (LSC) | 119755 | *IGS* | (SSC) | F |
| 39 | 44154 | *ycf3* | (LSC) | 138988 | *IGS* | (IRa) | P |
| 40 | 91805 | *ycf2* | (IRb) | 91841 | *ycf2* | (IRb) | F |
| 40 | 91805 | *ycf2* | (IRb) | 145963 | *ycf2* | (IRa) | P |
| 40 | 91841 | *ycf2* | (IRb) | 145999 | *ycf2* | (IRa) | P |
| 40 | 145963 | *ycf2* | (IRa) | 145999 | *ycf2* | (IRa) | F |
| 30 | 8320 | *trnS-GCU* | (LSC) | 45890 | *trnS-GGA* | (LSC) | P |
| 36 | 91803 | *ycf2* | (IRb) | 91821 | *ycf2* | (IRb) | F |
| 36 | 91803 | *ycf2* | (IRb) | 145987 | *ycf2* | (IRa) | P |
| 36 | 91821 | *ycf2* | (IRb) | 146005 | *ycf2* | (IRa) | P |
| 36 | 145987 | *ycf2* | (IRa) | 146005 | *ycf2* | (IRa) | F |
| 35 | 46999 | *IGS* | (LSC) | 46999 | *IGS* | (LSC) | P |
| 32 | 75724 | *petB* | (LSC) | 75724 | *petB* | (LSC) | P |
| 34 | 91823 | *ycf2* | (IRb) | 91841 | *ycf2* | (IRb) | F |
| 34 | 91823 | *ycf2* | (IRb) | 145969 | *ycf2* | (IRa) | P |
| 34 | 91841 | *ycf2* | (IRb) | 145987 | *ycf2* | (IRa) | P |
| 34 | 145969 | *ycf2* | (IRa) | 145987 | *ycf2* | (IRa) | F |
| 30 | 44166 | *ycf3* | (LSC) | 98829 | *IGS* | (IRb) | F |
| 30 | 44166 | *ycf3* | (LSC) | 138985 | *IGS* | (IRa) | P |
| 32 | 8318 | *trnS-GCU* | (LSC) | 35968 | *trnS-UGA* | (LSC) | F |
| 31 | 67915 | *IGS* | (LSC) | 67924 | *IGS* | (LSC) | F |
| 30 | 9909 | *trnG-UCC* | (LSC) | 36848 | *trnG-GCC* | (LSC) | F |
| 30 | 29970 | *IGS* | (LSC) | 30006 | *IGS* | (LSC) | P |
| 30 | 35970 | *trnS-UGA* | (LSC) | 45890 | *trnS-GGA* | (LSC) | P |
| 30 | 39079 | *psaB* | (LSC) | 41303 | *psaA* | (LSC) | F |
| 30 | 44166 | *ycf3* | (LSC) | 119767 | *ndhG* | (SSC) | F |
| 30 | 89391 | *ycf2* | (IRb) | 89433 | *ycf2* | (IRb) | F |
| 30 | 89391 | *ycf2* | (IRb) | 148381 | *ycf2* | (IRa) | P |
| 30 | 89433 | *ycf2* | (IRb) | 148423 | *ycf2* | (IRa) | P |
| 30 | 91804 | *ycf2* | (IRb) | 91858 | *ycf2* | (IRb) | F |
| 30 | 91804 | *ycf2* | (IRb) | 145956 | *ycf2* | (IRa) | P |
| 30 | 91833 | *ycf2* | (IRb) | 91851 | *ycf2* | (IRb) | F |
| 30 | 91833 | *ycf2* | (IRb) | 145963 | *ycf2* | (IRa) | P |
| 30 | 91851 | *ycf2* | (IRb) | 145981 | *ycf2* | (IRa) | P |
| 30 | 91858 | *ycf2* | (IRb) | 146010 | *ycf2* | (IRa) | P |
| 30 | 145956 | *ycf2* | (IRa) | 146010 | *ycf2* | (IRa) | F |
| 30 | 145976 | *ycf2* | (IRa) | 146012 | *ycf2* | (IRa) | F |
| 30 | 148381 | *ycf2* | (IRa) | 148423 | *ycf2* | (IRa) | F |

**Table S6.** *Verbascum sinaiticum* long repeat position table

| Repeat size (bp) | 1st start | location1 | | 2st start | location2 | | Repeat type |
| --- | --- | --- | --- | --- | --- | --- | --- |
| 41 | 98959 | *IGS* | (IRb) | 119951 | *ndhG* | (SSC) | F |
| 41 | 119951 | *ndhG* | (SSC) | 139196 | *IGS* | (IRa) | P |
| 44 | 75040 | *IGS* | (LSC) | 75040 | *IGS* | (LSC) | P |
| 39 | 44209 | *ycf3* | (LSC) | 98961 | *IGS* | (IRb) | F |
| 39 | 44209 | *ycf3* | (LSC) | 119953 | *ndhG* | (SSC) | F |
| 39 | 44209 | *ycf3* | (LSC) | 139196 | *IGS* | (IRa) | P |
| 40 | 91949 | *ycf2* | (IRb) | 91985 | *ycf2* | (IRb) | F |
| 40 | 91949 | *ycf2* | (IRb) | 146171 | *ycf2* | (IRa) | P |
| 40 | 91985 | *ycf2* | (IRb) | 146207 | *ycf2* | (IRa) | P |
| 40 | 146171 | *ycf2* | (IRa) | 146207 | *ycf2* | (IRa) | F |
| 30 | 248 | *IGS* | (LSC) | 248 | *IGS* | (LSC) | P |
| 30 | 8297 | *trnS-GCU* | (LSC) | 45926 | *trnS-GGA* | (LSC) | P |
| 35 | 42648 | *IGS* | (LSC) | 42675 | *IGS* | (LSC) | F |
| 36 | 91947 | *ycf2* | (IRb) | 91965 | *ycf2* | (IRb) | F |
| 36 | 91947 | *ycf2* | (IRb) | 146195 | *ycf2* | (IRa) | P |
| 36 | 91965 | *ycf2* | (IRb) | 146213 | *ycf2* | (IRa) | P |
| 36 | 146195 | *ycf2* | (IRa) | 146213 | *ycf2* | (IRa) | F |
| 32 | 47031 | *IGS* | (LSC) | 47031 | *IGS* | (LSC) | P |
| 34 | 91967 | *ycf2* | (IRb) | 91985 | *ycf2* | (IRb) | F |
| 34 | 91967 | *ycf2* | (IRb) | 146177 | *ycf2* | (IRa) | P |
| 34 | 91985 | *ycf2* | (IRb) | 146195 | *ycf2* | (IRa) | P |
| 34 | 146177 | *ycf2* | (IRa) | 146195 | *ycf2* | (IRa) | F |
| 30 | 44221 | *ycf3* | (LSC) | 98973 | *IGS* | (IRb) | F |
| 30 | 44221 | *ycf3* | (LSC) | 139193 | *IGS* | (IRa) | P |
| 32 | 8295 | *trnS-GCU* | (LSC) | 35956 | *trnS-UGA* | (LSC) | F |
| 30 | 9878 | *trnG-UCC* | (LSC) | 36850 | *trnG-GCC* | (LSC) | F |
| 30 | 29949 | *IGS* | (LSC) | 29985 | *IGS* | (LSC) | P |
| 30 | 35958 | *trnS-UGA* | (LSC) | 45926 | *trnS-GGA* | (LSC) | P |
| 30 | 38527 | *psaB* | (LSC) | 40742 | *psaA* | (LSC) | F |
| 30 | 39076 | *psaB* | (LSC) | 41300 | *psaA* | (LSC) | F |
| 30 | 44221 | *ycf3* | (LSC) | 119965 | *ndhG* | (SSC) | F |
| 30 | 89535 | *ycf2* | (IRb) | 89577 | *ycf2* | (IRb) | F |
| 30 | 89535 | *ycf2* | (IRb) | 148589 | *ycf2* | (IRa) | P |
| 30 | 89577 | *ycf2* | (IRb) | 148631 | *ycf2* | (IRa) | P |
| 30 | 91977 | *ycf2* | (IRb) | 91995 | *ycf2* | (IRb) | F |
| 30 | 91977 | *ycf2* | (IRb) | 146171 | *ycf2* | (IRa) | P |
| 30 | 91995 | *ycf2* | (IRb) | 146189 | *ycf2* | (IRa) | P |
| 30 | 146184 | *ycf2* | (IRa) | 146220 | *ycf2* | (IRa) | F |
| 30 | 148589 | *ycf2* | (IRa) | 148631 | *ycf2* | (IRa) | F |

**Table S7.** *Verbascum songaricum* long repeat position table

| Repeat size (bp) | 1st start | location1 | | 2st start | location2 | | Repeat type |
| --- | --- | --- | --- | --- | --- | --- | --- |
| 41 | 98799 | *IGS* | (IRb) | 118015 | *ndhA* | (SSC) | P |
| 41 | 118015 | *ndhA* | (SSC) | 138985 | *IGS* | (IRa) | F |
| 44 | 74863 | *IGS* | (LSC) | 74863 | *IGS* | (LSC) | P |
| 39 | 44092 | *ycf3* | (LSC) | 98801 | *IGS* | (IRb) | F |
| 39 | 44092 | *ycf3* | (LSC) | 118015 | *ndhA* | (SSC) | P |
| 39 | 44092 | *ycf3* | (LSC) | 138985 | *IGS* | (IRa) | P |
| 40 | 91789 | *ycf2* | (IRb) | 91825 | *ycf2* | (IRb) | F |
| 40 | 91789 | *ycf2* | (IRb) | 145960 | *ycf2* | (IRa) | P |
| 40 | 91825 | *ycf2* | (IRb) | 145996 | *ycf2* | (IRa) | P |
| 40 | 145960 | *ycf2* | (IRa) | 145996 | *ycf2* | (IRa) | F |
| 30 | 8282 | *trnS-GCU* | (LSC) | 45806 | *trnS-GGA* | (LSC) | P |
| 36 | 91787 | *ycf2* | (IRb) | 91805 | *ycf2* | (IRb) | F |
| 36 | 91787 | *ycf2* | (IRb) | 145984 | *ycf2* | (IRa) | P |
| 36 | 91805 | *ycf2* | (IRb) | 146002 | *ycf2* | (IRa) | P |
| 36 | 145984 | *ycf2* | (IRa) | 146002 | *ycf2* | (IRa) | F |
| 32 | 46911 | *IGS* | (LSC) | 46911 | *IGS* | (LSC) | P |
| 32 | 51505 | *ndhC* | (LSC) | 51505 | *ndhC* | (LSC) | R |
| 34 | 91807 | *ycf2* | (IRb) | 91825 | *ycf2* | (IRb) | F |
| 34 | 91807 | *ycf2* | (IRb) | 145966 | *ycf2* | (IRa) | P |
| 34 | 91825 | *ycf2* | (IRb) | 145984 | *ycf2* | (IRa) | P |
| 34 | 145966 | *ycf2* | (IRa) | 145984 | *ycf2* | (IRa) | F |
| 30 | 44104 | *ycf3* | (LSC) | 98813 | *IGS* | (IRb) | F |
| 30 | 44104 | *ycf3* | (LSC) | 138982 | *IGS* | (IRa) | P |
| 32 | 8280 | *trnS-GCU* | (LSC) | 35924 | *trnS-UGA* | (LSC) | F |
| 30 | 9873 | *trnG-UCC* | (LSC) | 36806 | *trnG-GCC* | (LSC) | F |
| 30 | 29927 | *IGS* | (LSC) | 29963 | *IGS* | (LSC) | P |
| 30 | 35926 | *trnS-UGA* | (LSC) | 45806 | *trnS-GGA* | (LSC) | P |
| 30 | 38481 | *psaB* | (LSC) | 40696 | *psaA* | (LSC) | F |
| 30 | 39030 | *psaB* | (LSC) | 41254 | *psaA* | (LSC) | F |
| 30 | 44104 | *ycf3* | (LSC) | 118012 | *ndhA* | (SSC) | P |
| 30 | 89375 | *ycf2* | (IRb) | 89417 | *ycf2* | (IRb) | F |
| 30 | 89375 | *ycf2* | (IRb) | 148378 | *ycf2* | (IRa) | P |
| 30 | 89417 | *ycf2* | (IRb) | 148420 | *ycf2* | (IRa) | P |
| 30 | 91817 | *ycf2* | (IRb) | 91835 | *ycf2* | (IRb) | F |
| 30 | 91817 | *ycf2* | (IRb) | 145960 | *ycf2* | (IRa) | P |
| 30 | 91835 | *ycf2* | (IRb) | 145978 | *ycf2* | (IRa) | P |
| 30 | 145973 | *ycf2* | (IRa) | 146009 | *ycf2* | (IRa) | F |
| 30 | 148378 | *ycf2* | (IRa) | 148420 | *ycf2* | (IRa) | F |

**Table S8.** *Verbascum thapsus* long repeat position table

| Repeat size (bp) | 1st start | location1 | | 2st start | location2 | | Repeat type |
| --- | --- | --- | --- | --- | --- | --- | --- |
| 52 | 57679 | *IGS* | (LSC) | 57730 | *IGS* | (LSC) | F |
| 41 | 98901 | *IGS* | (IRb) | 118144 | *ndhA* | (SSC) | P |
| 41 | 118144 | *ndhA* | (SSC) | 139116 | *IGS* | (IRb) | F |
| 44 | 74995 | *psbN* | (LSC) | 74995 | *psbN* | (LSC) | P |
| 39 | 44124 | *ycf3* | (LSC) | 98903 | *IGS* | (IRb) | F |
| 39 | 44124 | *ycf3* | (LSC) | 118144 | *ndhA* | (SSC) | P |
| 39 | 44124 | *ycf3* | (LSC) | 139116 | *IGS* | (IRb) | P |
| 40 | 91891 | *ycf2* | (IRb) | 91927 | *ycf2* | (IRb) | F |
| 40 | 91891 | *ycf2* | (IRb) | 146091 | *ycf2* | (IRa) | P |
| 40 | 91927 | *ycf2* | (IRb) | 146127 | *ycf2* | (IRa) | P |
| 40 | 146091 | *ycf2* | (IRa) | 146127 | *ycf2* | (IRa) | F |
| 30 | 8308 | *trnS-GCU* | (LSC) | 45847 | *IGS* | (LSC) | P |
| 36 | 91889 | *ycf2* | (IRb) | 91907 | *ycf2* | (IRb) | F |
| 36 | 91889 | *ycf2* | (IRb) | 146115 | *ycf2* | (IRa) | P |
| 36 | 91907 | *ycf2* | (IRb) | 146133 | *ycf2* | (IRa) | P |
| 36 | 146115 | *ycf2* | (IRa) | 146133 | *ycf2* | (IRa) | F |
| 34 | 91909 | *ycf2* | (IRb) | 91927 | *ycf2* | (IRb) | F |
| 34 | 91909 | *ycf2* | (IRb) | 146097 | *ycf2* | (IRa) | P |
| 34 | 91927 | *ycf2* | (IRb) | 146115 | *ycf2* | (IRa) | P |
| 34 | 146097 | *ycf2* | (IRa) | 146115 | *ycf2* | (IRa) | F |
| 30 | 44136 | *ycf3* | (LSC) | 98915 | *IGS* | (IRb) | F |
| 30 | 44136 | *ycf3* | (LSC) | 139113 | *IGS* | (IRb) | P |
| 30 | 46953 | *IGS* | (LSC) | 46953 | *IGS* | (LSC) | P |
| 32 | 8306 | *trnS-GCU* | (LSC) | 35943 | *trnS-UGA* | (LSC) | F |
| 30 | 9907 | *IGS* | (LSC) | 36824 | *trnG-GCC* | (LSC) | F |
| 30 | 29945 | *IGS* | (LSC) | 29981 | *IGS* | (LSC) | P |
| 30 | 35945 | *trnS-UGA* | (LSC) | 45847 | *IGS* | (LSC) | P |
| 30 | 38498 | *psaB* | (LSC) | 40713 | *psaA* | (LSC) | F |
| 30 | 39047 | *psaB* | (LSC) | 41271 | *psaA* | (LSC) | F |
| 30 | 44136 | *ycf3* | (LSC) | 118141 | *ndhA* | (SSC) | P |
| 30 | 91919 | *ycf2* | (IRb) | 91937 | *ycf2* | (IRb) | F |
| 30 | 91919 | *ycf2* | (IRb) | 146091 | *ycf2* | (IRa) | P |
| 30 | 91937 | *ycf2* | (IRb) | 146109 | *ycf2* | (IRa) | P |
| 30 | 146104 | *ycf2* | (IRa) | 146140 | *ycf2* | (IRa) | F |

**Table S9:** SRRs present in *Verbascum* species.

| **SSR number.** | **SSR type** | **SSR** | **size** | **start** | **end** | **location** | **Region** |
| --- | --- | --- | --- | --- | --- | --- | --- |
| 1 | p1 | (A)11 | 11 | 4791 | 4801 | IGS (*trnK-UUU*-*rps*16) | LSC |
| 2 | p1 | (T)10 | 10 | 8027 | 8036 | IGS *(psbK-psbI*) | LSC |
| 3 | p1 | (A)10 | 10 | 8271 | 8280 | IGS *(psbI-trns-GCU)* | LSC |
| 4 | p2 | (TA)6 | 12 | 8454 | 8465 | IGS (*trnS-GSU-trnG-UCC)* | LSC |
| 5 | p1 | (A)12 | 12 | 8701 | 8712 | IGS (*trnS-GSU-trnG-UCC*) | LSC |
| 6 | p1 | (T)11 | 11 | 8902 | 8912 | IGS (*trnS-GSU-trnG-UCC*) | LSC |
| 7 | p1 | (T)12 | 12 | 13212 | 13223 | IGS (*atpF-atpH*) | LSC |
| 8 | p1 | (T)10 | 10 | 14592 | 14601 | IGS *(atpH-atpI*) | LSC |
| 9 | c | (T)10 | 23 | 16455 | 16477 | IGS (*rps*2-*rpoc*2) | LSC |
| 10 | p1 | (T)11 | 11 | 18670 | 18680 | *rpoc*2 | LSC |
| 11 | p1 | (A)10 | 10 | 27400 | 27409 | IGS (*rpoB-trnc-GCA*) | LSC |
| 12 | p1 | (T)11 | 11 | 36892 | 36902 | IGS (*trnG-GCC-trnfm-CAU*) | LSC |
| 13 | p1 | (A)10 | 10 | 44859 | 44868 | *ycf3* | LSC |
| 14 | p1 | (A)10 | 10 | 45113 | 45122 | IGS (*ycf3*-*trnS-GGA*) | LSC |
| 15 | p1 | (A)12 | 12 | 45667 | 45678 | IGS (*ycf3-trnS-GGA*) | LSC |
| 16 | p1 | (T)10 | 10 | 46894 | 46903 | IGS (*rps*4*-trnT-UGU*) | LSC |
| 17 | c | (TA) | 30 | 47033 | 47062 | IGS (*rps*4-*trnT-UGU*) | LSC |
| 18 | p1 | (A)10 | 10 | 47698 | 47707 | IGS (*trnT-UGU*-*trnL-UAA*) | LSC |
| 19 | p1 | (T)13 | 13 | 48300 | 48312 | *trnL-UAA* | LSC |
| 20 | p1 | (T)11 | 11 | 49173 | 49183 | IGS (*trnF-GAA-ndhJ)* | LSC |
| 21 | c | (T)10 | 69 | 55400 | 55468 | *atpB-rbcL* | LSC |
| 22 | p1 | (G)12 | 12 | 61431 | 61442 | IGS (*ycf*4*-cemA*) | LSC |
| 23 | p1 | (T)10 | 10 | 64444 | 64453 | IGS (petA-psbJ) | LSC |
| 24 | p2 | (TA)6 | 12 | 67616 | 67627 | IGS (*trnP-UGG-psaJ*) | LSC |
| 25 | p1 | (A)11 | 11 | 69211 | 69221 | IGS (r*ps*18*-rpl*20) | LSC |
| 26 | p1 | (A)11 | 11 | 69957 | 69967 | IGS ( *rpl*20-*rp*12) | LSC |
| 27 | p1 | (T)10 | 10 | 70778 | 70787 | IGS *(rpl2-clpP)* | LSC |
| 28 | p1 | (A)10 | 10 | 72741 | 72750 | IGS (c*lpP-psbB*) | LSC |
| 29 | p1 | (C)10 | 10 | 74829 | 74838 | IGS *(psbB-psbT)* | LSC |
| 30 | p1 | (T)13 | 13 | 75893 | 75905 | petB | LSC |
| 31 | p1 | (T)10 | 10 | 78838 | 78847 | *rpoA* | LSC |
| 32 | p1 | (T)11 | 11 | 80738 | 80748 | IGS (*infA-rps8*) | LSC |
| 33 | p1 | (T)10 | 10 | 81240 | 81249 | IGS (*rps8-rpl14*) | LSC |
| 34 | p1 | (A)10 | 10 | 82480 | 82489 | *rpl*16 | LSC |
| 35 | p1 | (T)13 | 13 | 103133 | 103145 | *trnI-GAU* | IRb |
| 36 | p1 | (A)11 | 11 | 113277 | 113287 | IGS *(rpl32-trnL-UAG*) | SSC |
| 37 | p1 | (A)10 | 10 | 113721 | 113730 | *trnL-UAG* | SSC |
| 38 | p1 | (A)10 | 10 | 116968 | 116977 | IGS (*ndhB-psaC*) | SSC |
| 39 | p2 | (TA)6 | 12 | 123447 | 123458 | IGS *(rps15-ycf1)* | SSC |
| 40 | p1 | (T)11 | 11 | 126039 | 126049 | *ycf1* | SSC |
| 41 | p1 | (T)10 | 10 | 127974 | 127983 | *ycf1* | ssc |
| 42 | p1 | (A)13 | 13 | 135052 | 135064 | *trnI-GAU* | IRa |

**Table S10**: Total number of SSRs repeats

| SSRs | *V. sinaiticum* | *V .brevipedicellatum* | *V. thapsus* | *V. blattaria* | *V. chaixii* | *V. phoeniceum* | *V. songaricum* |
| --- | --- | --- | --- | --- | --- | --- | --- |
| A/T | 37 | 31 | 38 | 31 | 32 | 33 | 38 |
| C/G | 2 | 0 | 1 | 0 | 2 | 0 | 0 |
| AT/AT | 7 | 5 | 6 | 8 | 7 | 7 | 7 |
| AAT/ATT | 1 | 1 | 1 | 1 | 1 | 1 | 2 |
| AAC/GTT | 0 | 0 | 0 | 0 | 0 | 1 | 0 |
| AAAG/CTTT | 1 | 3 | 1 | 1 | 2 | 2 | 1 |
| AAAT/ATTT | 1 | 1 | 2 | 2 | 1 | 1 | 1 |
| AACT/AGTT | 2 | 2 | 2 | 0 | 2 | 2 | 2 |
| ACAG/CTGT | 1 | 1 | 1 | 1 | 1 | 1 | 1 |
| AGAT/ATCT | 1 | 1 | 1 | 1 | 1 | 1 | 1 |
| ACCTAT/AGGTAT | 0 | 0 | 0 | 1 | 0 | 0 | 0 |
| Total no. of SSRs | 53 | 45 | 53 | 46 | 49 | 49 | 53 |
