# Supplementary material for: Taxonomy, comparative genomics of Mullein (Verbascum, Scrophulariaceae), with implications for the evolution of Verbascum and Lamiales
Source: BMC Genomics. 2022 Aug 8;23:566. doi: 10.1186/s12864-022-08799-9 (PMC9358837; doi:10.1186/s12864-022-08799-9)
Supplement: Supplementary file 1 — Additional file 1: Fig. S1. mVISTA percentage identity comparison among five plastomes from Verbascum using V. sinaiticum as reference. X-axis indicates the sequence coordinates in the whole cp genome. Y-axis represents the similarity of the aligned regions, indicating percent identity to the reference genome (50-100%). [file 12864_2022_8799_MOESM1_ESM.pdf]

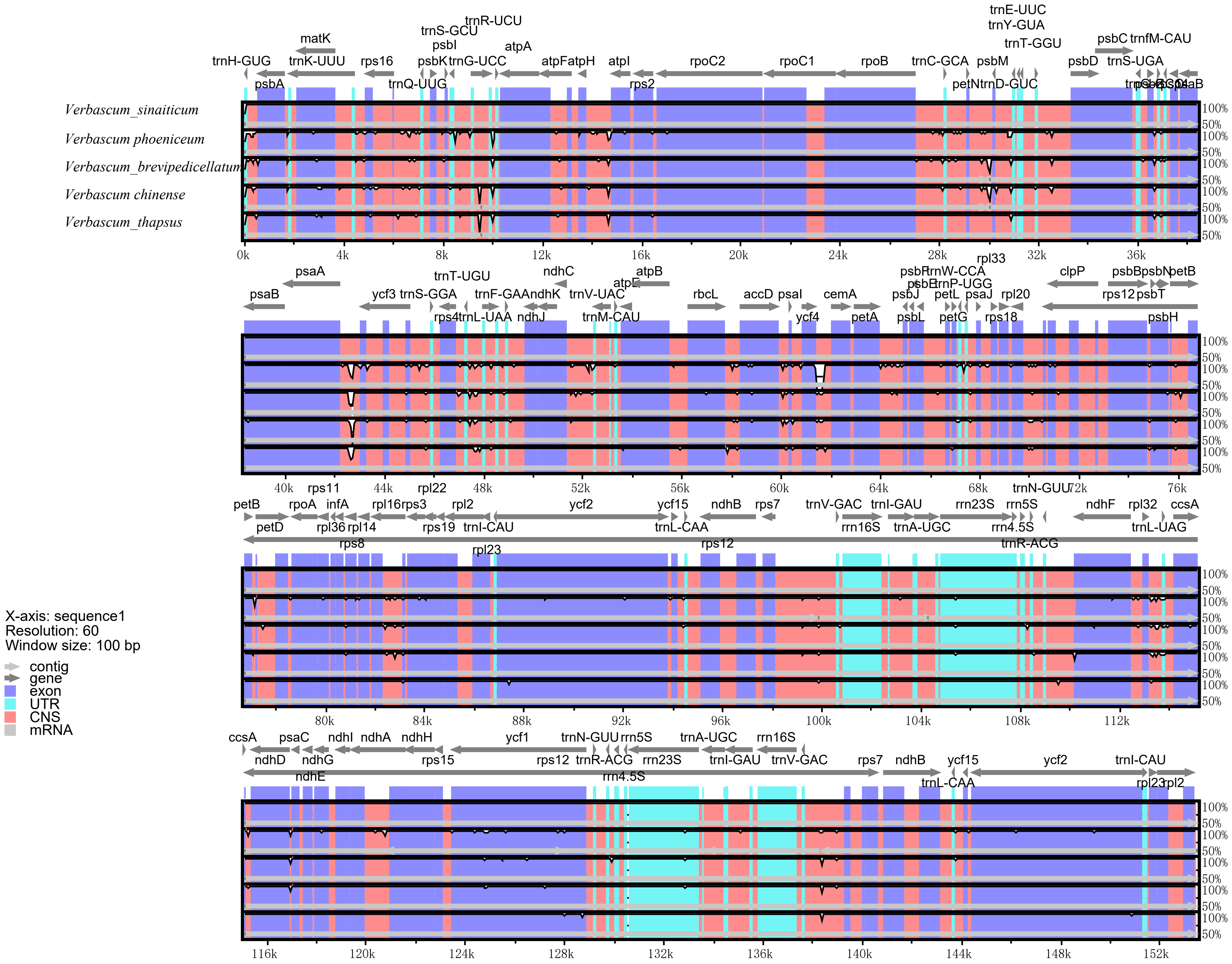

Verbascum_sinaiticum
Verbascum phoeniceum
Verbascum_brevipedicellatum
Verbascum chinense
Verbascum_thapsus
X-axis: sequence1
Resolution: 60
Window size: 100 bp
contig
gene
exon
UTR
CNS
mRNA
100%
50%
0k
4k
8k
12k
16k
20k
24k
28k
32k
36k
40k
44k
48k
52k
56k
60k
64k
68k
72k
76k
80k
84k
88k
92k
96k
100k
104k
108k
112k
116k
120k
124k
128k
132k
136k
140k
144k
148k
152k
trnH-GUG
psbA
matK
trnK-UUU
rps16
trnQ-UUG
trnS-GCU
psbI
psbK
trnR-UCU
trnG-UCC
atpA
atpF
atpH
atpI
rps2
rpoC2
rpoC1
rpoB
trnC-GCA
petN
psbM
trnD-GUC
trnE-UUC
trnY-GUA
trnT-GGU
psbD
psbC
trnS-UGA
trnfM-CAU
psaB
psaA
ycf3
trnS-GGA
rps4
trnT-UGU
trnL-UAA
trnF-GAA
ndhJ
ndhK
ndhC
trnV-UAC
trnM-CAU
atpE
atpB
rbcL
accD
psaI
ycf4
cemA
petA
psbJ
psbL
psbF
psbE
trnW-CCA
trnP-UGG
petL
petG
psaJ
rpl33
rps18
rpl20
clpP
psbB
psbT
psbN
psbH
petB
petD
rpoA
rps11
rpl36
infA
rps8
rpl14
rpl16
rps3
rpl22
rps19
rpl2
rpl23
trnI-CAU
ycf2
ycf15
trnL-CAA
ndhB
rps7
rps12
trnV-GAC
rrn16S
trnI-GAU
trnA-UGC
rrn23S
rrn4.5S
rrn5S
trnR-ACG
trnN-GUU
ndhF
rpl32
trnL-UAG
ccsA
ndhD
psaC
ndhE
ndhG
ndhI
ndhA
ndhH
rps15
ycf1
